# Integrative Neuroimmune Role of the Parasympathetic Nervous System, Vagus Nerve and Gut Microbiota in Stress Modulation: A Narrative Review

**DOI:** 10.3390/ijms262311706

**Published:** 2025-12-03

**Authors:** Natalia Kurhaluk, Renata Kołodziejska, Piotr Kamiński, Halina Tkaczenko

**Affiliations:** 1 Institute of Biology, Pomeranian University in Słupsk, Arciszewski St. 22 B, 76-200 Słupsk, Poland; halina.tkaczenko@upsl.edu.pl; 2 Department of Medical Biology and Biochemistry, Collegium Medicum in Bydgoszcz, Nicholaus Copernicus University, M. Karłowicz St. 24, 85-092 Bydgoszcz, Poland; renatak@cm.umk.pl; 3 Department of Medical Biology and Biochemistry, Division of Ecology and Environmental Protection, Collegium Medicum in Bydgoszcz, Nicolaus Copernicus University in Toruń, M. Skłodowska-Curie St. 9, 85-094 Bydgoszcz, Poland; 4 Department of Biotechnology, Institute of Biological Sciences, Faculty of Biological Sciences, University of Zielona Góra, Prof. Z. Szafran St. 1, 65-516 Zielona Góra, Poland

**Keywords:** parasympathetic nervous system, vagus nerve, gut-brain axis, microbiota-gut-brain communication, dysbiosis, vagal tone

## Abstract

It has been demonstrated that prolonged exposure to stress engenders a plethora of neuropsychiatric, immune and metabolic disorders. However, its pathophysiology transcends the conventional hypothalamic–pituitary–adrenal (HPA) axis. This review addresses the central question of how integrated neural and microbial pathways regulate stress responses and resilience. We present a model in which the parasympathetic nervous system (particularly the vagus nerve) and the gut microbiota interact to form a bidirectional neuroimmune network that modulates the HPA axis, immune function, neurotransmitter balance, and metabolic adaptation. Key molecular pathways include nitric oxide synthesis via the classical nitric oxide synthase (NOS)-dependent and microbiota-mediated nitrate–nitrite routes, inducible nitric oxide synthase (iNOS) regulation, nuclear factor erythroid 2-related factor 2 (Nrf2) signalling, lysosomal function, autophagy and the cholinergic anti-inflammatory reflex. Other pathways include the gamma-aminobutyric acid (GABA) and serotonin (5-HT) systems, NF-κB (nuclear factor kappa-light-chain-enhancer of activated B cells) signalling, polyamine metabolism and peroxisome proliferator-activated receptor gamma (PPARγ). Intermittent hypoxia training (IHT) enhances mitochondrial function, oxidative stress responses, autonomic balance and gut microbiota composition. This promotes parasympathetic activity and stress resilience that is tailored to the individual. These adaptations support the concept of personalised stress response profiles based on hypoxic adaptability. Clinical implications include combining IHT with vagus nerve stimulation, probiotics, dietary strategies, and stress reduction techniques. Monitoring vagal tone and microbiota composition could also serve as predictive biomarkers for personalised interventions in stress-related disorders. This integrative framework highlights the therapeutic potential of targeting the parasympathetic system and the gut microbiota to modulate stress.

## 1. Introduction

Chronic stress is a pervasive and complex physiological condition that has far-reaching effects on multiple systems, including the nervous, immune, endocrine, and gastrointestinal systems [1]. Unlike acute stress, which plays an adaptive role in survival, chronic stress has a detrimental effect on cellular homeostasis and systemic function. Prolonged activation of the hypothalamic–pituitary-adrenal (HPA) axis results in sustained elevation of glucocorticoids, such as cortisol. This contributes to immunosuppression, neurodegeneration, mitochondrial dysfunction, and dysbiosis of the gut microbiome [2]. These alterations not only compromise immediate physiological resilience but also create long-term vulnerability to chronic inflammatory and degenerative disorders.

Building on this, Gershon and Margolis [3] and Chang et al. [4] have broadened the traditional view of stress as a neuroendocrine response to include the gut–brain axis. They emphasise the critical roles of the enteric nervous system and gut microbiota in shaping systemic and neural stress responses. Meanwhile, Claudino Dos Santos et al. (2023) demonstrated that the parasympathetic nervous system (PSNS), particularly the vagus nerve, acts as a key hub for two-way communication between the brain and visceral organs [5]. This modulates not only cardiovascular and digestive functions but also has significant immunomodulatory and anti-inflammatory effects. This has been observed in cases such as liver tumours [6]. Together, these findings highlight the importance of considering neural, immune, and microbial factors in stress biology, as vagal signalling is now recognised as a critical factor in determining resilience or vulnerability to stress-related pathology.

In addition to these neural mechanisms, Wessler and Kirkpatrick (2008) demonstrated that all components of the cholinergic system, including acetylcholine (ACh), nicotinic and muscarinic receptors, and acetylcholinesterase, are present in non-neuronal cells, such as epithelial, endothelial, immune and stem cells [7]. In these contexts, ACh regulates processes such as proliferation, differentiation, barrier formation, migration, and ion and water transport through distinct receptor patterns [7]. This reveals a novel, independent layer of cellular regulation that is functionally comparable to classical neurotransmission. These findings emphasise the potential of the non-neuronal cholinergic system as a novel therapeutic target for stress, inflammation, and disease, connecting cellular and systemic levels of adaptation.

In addition to neurochemical signalling, advances in microbiome science and neuroimmunology have emphasised the importance of microbial diversity and metabolite production in shaping the stress response [8]. Microbial products, such as short-chain fatty acids (SCFAs), neurotransmitter precursors and tryptophan catabolites, influence brain function and behaviour via neural and humoral pathways. Perturbations in this dynamic network have been linked to a range of stress-related pathologies, including anxiety, depression, irritable bowel syndrome and systemic inflammation [9]. Reduced microbial diversity and impaired SCFA production are increasingly being recognised as biomarkers of stress-related disorders, offering promising diagnostic and therapeutic prospects.

A growing body of evidence suggests that the dysregulation of the PSNS (specifically, vagal tone) in conjunction with disturbances to the gut microbiome plays a significant role in the pathophysiology of various chronic diseases, as demonstrated by Han et al. [10] and Tan et al. [11]. These diseases include neuropsychiatric, gastrointestinal, metabolic, cardiovascular and immune-mediated disorders [12]. The PSNS, via its main efferent nerve, the vagus nerve, modulates heart rate, digestive function, inflammation, neurotransmission and gut–brain communication [13]. Concurrently, the intestinal microbiota exerts a profound influence on host physiology via microbial metabolites such as SCFAs, neurotransmitter analogues (e.g., GABA and serotonin) and immune-regulatory molecules. Disruption to this finely tuned interplay between vagal signalling and microbiota-derived mediators is now recognised as a central pathogenic mechanism linking stress to chronic disease. Dysregulation of these two systems, whether independently or through disrupted bidirectional signalling, can trigger a cascade of maladaptive responses [14].

A comprehensive literature search was conducted across four major databases: PubMed, Scopus, Web of Science and Embase. The search covered publications from January 2005 to March 2025 in order to capture the rapid evolution of microbiota–gut–brain axis research and advances in neuroimmunology. The search was limited to peer-reviewed original research articles, meta-analyses and systematic reviews. Articles were excluded if they were written in a language other than English, were not available in full text, were opinion pieces, or focused solely on sympathetic or enteric pathways without addressing the parasympathetic nervous system or vagal involvement. All retrieved articles were independently screened by all authors, with any discrepancies resolved through discussion. Studies were selected based on clear evidence of interaction or dysregulation between the parasympathetic system and the microbiome in the context of disease. The following keywords were used: “parasympathetic nervous system”, “vagus nerve”, “gut-brain axis”, “microbiota-gut-brain communication”, “dysbiosis”, “vagal tone”, “neuroinflammation”, and “inflammatory reflex”. Following revisions in response to reviewer comments, the bibliography was expanded to include a total of 258 references, reflecting the comprehensive and integrative nature of this review.

The primary objective of this review is to provide an integrative, mechanistic synthesis of existing knowledge on the interaction between the parasympathetic nervous system (particularly vagal activity) and the gut microbiome in the context of chronic stress and its pathophysiological consequences. By integrating findings from neurobiology, immunology, microbiome science and clinical physiology, this study aims to elucidate how modulation of these two systems can influence systemic resilience to stress and potentially reverse or reduce stress-related disorders.

This study is particularly pertinent in light of the growing recognition that conventional models of stress pathophysiology, which focus primarily on the HPA axis, are unable to fully account for the significant interindividual variability observed in stress responses and disease susceptibility [15,16]. Accordingly, there is an urgent need to move beyond HPA-centric frameworks and incorporate parasympathetic and microbiota-related mechanisms to gain a fuller understanding of stress biology. Recent findings on alternative nitric oxide synthesis pathways, vagus-mediated anti-inflammatory signalling and microbiota-derived neuromodulators highlight the molecular depth and complexity of this bidirectional interaction.

The microbiota–gut–brain axis, with the vagus nerve acting as a central bidirectional conduit, offers a more nuanced and systemic approach to understanding how stress is perceived, processed, and manifested physiologically and behaviourally. This concept has been demonstrated in previous studies by the Bonaz team [17,18]. Complementary insights from molecular studies demonstrate how alternative nitric oxide synthesis pathways [19], vagus-mediated anti-inflammatory mechanisms and microbiota-derived neuromodulators contribute to stress modulation at cellular and systemic levels [20,21]. This convergence of clinical and molecular evidence highlights the translational potential of targeting the vagus-microbiome axis in therapeutic strategies.

This review is timely in focusing on the dual and interactive roles of the parasympathetic system and the microbiome in shaping adaptive and maladaptive responses to stress. While these domains have been extensively studied in isolation, relatively few studies have synthesised them into a unified conceptual framework that considers practical potential. The review emphasises the therapeutic implications of modulating vagal tone and microbiome composition through lifestyle, pharmacological, dietary or neuromodulatory interventions as a non-invasive, personalised approach to mitigating the physiological and psychological burdens of stress-related diseases [22].

The review is strengthened by its consideration of the rapidly evolving nature of microbiome and vagus nerve research, and of the ongoing mental health crisis exacerbated by global stressors such as the ongoing effects of the COVID-19 pandemic and climate-related uncertainty [23,24]. Moreover, the inclusion of precision-medicine approaches, such as microbiome profiling and vagus nerve stimulation technologies, positions this review at the interface of basic science and clinical application, making it highly relevant in addressing an urgent societal and medical need. The present study aligns with contemporary trends in systems biology and precision medicine by advocating a paradigm shift towards treating the ‘neuro-microbiological interface’ as a key axis in human health and disease prevention.

## 2. Physiological and Molecular Basis of Parasympathetic Activity

### 2.1. Parasympathetic Regulation of Systemic Homeostasis: Inflammation, Metabolism, and Adaptation

The parasympathetic nervous system (PSNS) is one of the two main divisions of the autonomic nervous system (ANS). It operates in a complementary and often antagonistic manner to the sympathetic nervous system [25]. Its main functions are to conserve energy and maintain long-term physiological stability, which are often referred to as the ‘rest and digest’ functions.

Anatomically, parasympathetic outflow originates in the brainstem and sacral spinal cord. Cranial parasympathetic fibres emerge from cranial nerves III (oculomotor), VII (facial), IX (glossopharyngeal) and, most notably, X (vagus nerve), the latter of which innervates a broad range of thoracic and abdominal organs. Sacral outflow (S2–S4) innervates pelvic structures such as the bladder, colon and reproductive organs [25]. Unlike the sympathetic system, the location of parasympathetic ganglia in close proximity to or within the effector organs enables precise, localised control of physiological processes [26].

**Figure 1:**
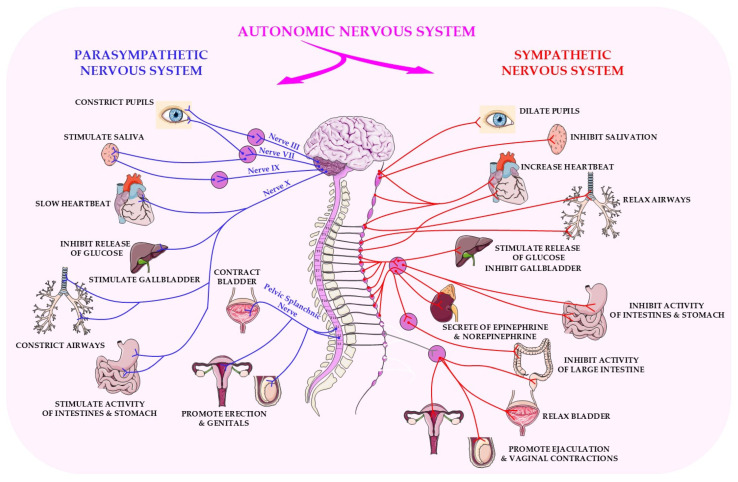
**The autonomic nervous system.** The autonomic nervous system comprises two functionally distinct branches: the sympathetic and the parasympathetic nervous systems. These branches exert opposing physiological effects in order to maintain internal balance. The sympathetic nervous system is activated in response to stress or danger, preparing the body for ‘fight or flight’. This results in pupil dilation, an increased heart rate and cardiac output, relaxed airways, dilated blood vessels in skeletal muscles, reduced gastrointestinal motility and restricted blood flow to the digestive organs. This state enables the body to respond rapidly and efficiently to external threats and aids recovery from stressful events. In contrast, the parasympathetic nervous system promotes ‘rest and digest’ functions, supporting recovery, energy conservation and homeostasis. It causes pupil constriction, slows the heart rate, narrows the airways, enhances intestinal peristalsis, stimulates the secretion of digestive enzymes and saliva, promotes glycogen storage in the liver and facilitates nutrient absorption. Together, these systems dynamically balance the body’s physiological responses to external and internal stimuli. **Abbreviations:** Nerve III—cranial nerve (perimotor); Nerve VII—facial nerve; Nerve IX—glossopharyngeal nerve; Nerve X—vagus nerve. This figure was created using Servier Medical Art (available at https://smart.servier.com/) (accessed on 1 May 2025).

As shown in Figure 1, the autonomic nervous system comprises two functionally distinct branches: the sympathetic and parasympathetic systems. These branches exert opposing physiological effects in order to maintain internal homeostasis. The figure illustrates the distinct organisation of parasympathetic projections in comparison to sympathetic pathways.

It is well documented that ACh is the primary neurotransmitter of the PSNS [27]. ACh is synthesised from choline and acetyl-CoA by the enzyme choline acetyltransferase [28]. The release of ACh by preganglionic fibres activates nicotinic receptors on postganglionic neurons. Conversely, postganglionic fibres release ACh, which stimulates muscarinic receptors (predominantly M2 and M3 subtypes) on target tissues [29,30]. M2 receptors mediate cardiac inhibition, while M3 receptors stimulate smooth muscle contraction and glandular secretion [31]. Additional muscarinic subtypes (e.g., M1 and M4) are also expressed in the central nervous system, linking parasympathetic signalling to higher-order processes such as cognition, arousal and emotional regulation [32].

In addition to cholinergic signalling, several co-transmitters, including nitric oxide (NO), vasoactive intestinal peptide (VIP) and adenosine triphosphate (ATP), have been identified as modulators of parasympathetic tone, particularly within the gastrointestinal and vascular systems. These secondary mediators contribute to the fine-tuning of organ-specific responses, including vasodilation, immune regulation and secretomotor activity [33,34]. Recent evidence also implicates endocannabinoids and neuropeptides (e.g., substance P and neuropeptide Y) as modulators of parasympathetic signalling, thus broadening the spectrum of molecules involved in systemic homeostatic regulation [35].

Parasympathetic activation induces a variety of anabolic and restorative responses that are vital for maintaining homeostasis. These include bradycardia (a reduced heart rate), decreased myocardial contractility, bronchoconstriction, increased gastrointestinal motility and secretion, salivation, lacrimation and urinary bladder contraction [36]. In contrast, sympathetic activation prioritises immediate survival through energy mobilisation. The vagus nerve exerts a particular influence over autonomic control in the heart, lungs, stomach, pancreas, intestines, liver and kidneys. This ensures that metabolic processes, digestion and recovery mechanisms are increased during non-stressful states, thereby contributing to long-term physiological balance [25].

Recent research has provided compelling evidence that the PSNS plays a pivotal role in orchestrating immunological and metabolic processes [31]. Beyond its classical autonomic functions, the PSNS has emerged as a key regulator of immunomodulatory and stress-responsive mechanisms, as previously demonstrated by Pavlov and Tracey [37]. A central feature of these processes is the “inflammatory reflex”, which is defined as a neuroimmune circuit in which the vagus nerve detects peripheral inflammatory signals and modulates systemic cytokine production via cholinergic signalling pathways [38,39]. The functional integrity of this reflex is increasingly recognised as a determinant of susceptibility to chronic inflammatory and metabolic diseases. This highlights the therapeutic relevance of targeting vagal tone through neuromodulation, biofeedback and microbiome-oriented interventions [18,40].

A key component of the cholinergic anti-inflammatory pathway is the α7 subunit of the nicotinic acetylcholine receptor (α7nAChR). Identifying this subunit has significantly advanced our understanding of neuroimmune regulation of inflammation [41]. Experimental evidence suggests that activating the α7nAChR is essential to inhibit the release of pro-inflammatory cytokines, particularly tumour necrosis factor (TNF), in response to vagus nerve stimulation [42]. This mechanism plays a pivotal role in preserving immune homeostasis and preventing excessive inflammatory responses [39]. These findings establish a direct and functional link between neural signalling and immune modulation, offering a novel conceptual framework through which the central nervous system may influence peripheral immune function. Given the well-documented link between chronic psychological and physiological stress and elevated systemic inflammation, elucidating this pathway highlights its potential as a therapeutic target for stress-related disorders. Specifically, interventions that stimulate the α7nAChR, either pharmacologically or via vagus nerve stimulation, have shown promise in reducing the inflammatory effects of stress. This contributes to the development of targeted anti-inflammatory and stress-relieving strategies [37].

The importance of the α7nAChR in immune regulation is reinforced by its predominant expression on immune cells, particularly macrophages, as demonstrated by Pechlivanidou et al. [43]. These findings contribute to our broader understanding of the non-neuronal cholinergic system as a critical modulator of immune function. Within this system, ACh is synthesised by CD4+ T cells through the enzymatic activity of choline acetyltransferase. ACh acts in an autocrine and paracrine fashion on both muscarinic and nicotinic receptors, with a notable role attributed to α7nAChRs. This cholinergic signalling results in the suppression of pro-inflammatory cytokine production and the promotion of immune homeostasis [44]. The discovery of SLURP-1, an endogenous α7nAChR ligand produced by dendritic cells that enhances ACh synthesis and supports T cell development, further illustrates the complexity and importance of this cholinergic network in immune modulation [45]. Moreover, recent studies have highlighted the interaction between α7nAChR signalling and the intracellular JAK2/STAT3 and NF-κB pathways. This provides insight into how cholinergic modulation directly regulates the transcription of inflammatory mediators [46]. Collectively, these findings emphasise the multifaceted role of cholinergic signalling. They also highlight its potential as a therapeutic target for inflammatory and stress-related conditions, as well as its function as a bridge between the nervous and immune systems.

Vagal stimulation suppresses the release of pro-inflammatory cytokines such as TNF-α and IL-1β, thereby linking neural activity to immune tolerance and the control of systemic inflammation [47]. The parasympathetic nervous system maintains mitochondrial homeostasis, modulates oxidative stress, and preserves gut epithelial integrity—all of which are essential processes for metabolic regulation and resilience to chronic stress [48]. Together, these mechanisms establish the vagus nerve as a central regulator at the intersection of neuroimmunology and gastroenterology, integrating inflammatory, metabolic, and barrier functions [40].

Much research has focused on the functional relevance and clinical implications of the subject. In particular, the PSNS has been identified as a critical factor in determining an individual’s physiological resilience and adaptive capacity [49]. Numerous clinical conditions have been linked to impaired parasympathetic tone, including depression, anxiety, irritable bowel syndrome (IBS), cardiovascular diseases, type 2 diabetes and autoimmune disorders [50,51]. Heart rate variability, a reliable, non-invasive proxy for vagal activity, is widely used to evaluate autonomic balance and stress vulnerability. Low heart rate variability is now considered an independent predictor of morbidity and mortality, further reinforcing its diagnostic and prognostic value in clinical practice [52]. Therapeutic strategies targeting parasympathetic enhancement, such as vagus nerve stimulation (VNS), biofeedback, diaphragmatic breathing, mindfulness-based interventions and microbiota modulation, are gaining recognition for their potential to restore autonomic equilibrium and improve clinical outcomes [53,54]. Thus, a thorough understanding of parasympathetic physiology is critical for advancing integrative medicine and developing personalised, non-pharmacological interventions for treating chronic stress-related diseases [55]. This integrated perspective suggests that enhancing vagal tone and α7nAChR signalling may represent convergent therapeutic strategies that combine neural modulation with immunoregulation to promote systemic resilience.

### 2.2. The Vagus Nerve: A Central Hub Linking Neural, Immune, and Microbial Networks

A plethora of studies have emphasised that the vagus nerve (cranial nerve X) is the main efferent pathway of the parasympathetic nervous system. It innervates many visceral organs, such as the heart, lungs, gastrointestinal tract, pancreas, liver and kidneys [56]. Its dual functionality, comprising afferent (sensory) and efferent (motor) fibres, enables bidirectional communication between the central nervous system (CNS) and peripheral tissues [57]. The majority of these fibres (around 80%) are afferent, transmitting signals from peripheral organs to the brainstem, particularly the nucleus tractus solitarius [58]. From there, signals are relayed to higher-order centres, such as the hypothalamus, amygdala and prefrontal cortex, where they integrate autonomic, endocrine and behavioural responses. This extensive afferent network is crucial for understanding the role of the vagus nerve as a central mediator of the gut–brain axis. It links peripheral organ states to central processes such as mood regulation, cognition, and emotional resilience [18,58,59]. The anatomical and functional complexity of this system establishes the vagus nerve as a vital interface in regulating systemic homeostasis, especially in situations involving stress, inflammation, and microbial disturbance [60].

The role of neuroimmune modulation via the cholinergic anti-inflammatory pathway continues to attract considerable attention, with a growing body of evidence highlighting its clinical and physiological relevance [61]. The vagus nerve is a central focus of this research. Its immunomodulatory capacity via the cholinergic anti-inflammatory pathway (CAP) has been studied in detail. In this reflex circuit, vagal afferents detect pro-inflammatory cytokines and pathogen-associated molecular patterns (PAMPs). This activates efferent vagal signalling pathways, which dampen inflammatory responses and restore immune balance. Importantly, α7nAChRs expressed on macrophages and dendritic cells are critical effectors of this pathway, translating neural input into cytokine suppression [37,38].

A study by Giri et al. (2019) showed that mice lacking gut microbiota had significantly impaired epinephrine release in response to hypoglycaemic stress [62]. This suggests that early-life microbial colonisation is essential for proper sympathoadrenal stress response development. Building on this, LaGamma et al. (2021) showed that oral supplementation with short-chain fatty acids—key microbial metabolites—can restore this impaired response following antibiotic-induced depletion of the microbiota, highlighting the therapeutic potential of targeting the gut microbiome to support stress regulation [63]. These findings integrate the microbiota into the broader vagal regulatory network, suggesting that microbial metabolites act not only locally in the gut but also systemically via vagus-mediated neuroimmune and neuroendocrine pathways [10,64].

### 2.3. Non-Neuronal Acetylcholine and Cell Function

Studies by Wessler and Kirkpatrick [8,65,66] and Mashimo et al. [44] have demonstrated that all components of the cholinergic system—including choline acetyltransferase (ChAT), the mechanisms of acetylcholine synthesis and release, and receptors—are functionally expressed in non-neuronal cells, independently of cholinergic innervation. There, they modulate diverse cellular functions by activating nicotinic and muscarinic ACh receptors. These receptors trigger multiple interconnected signalling pathways, including PI3K/Akt (energy metabolism, proliferation, apoptosis and migration), BAD (pro-apoptotic signalling), intracellular calcium/CaMKII (calcium-dependent responses), DAG/IP3 (phospholipase C-dependent signalling), MAPK/ERK (proliferation and differentiation), Jak/STAT (transcriptional regulation), PKA/PKC/PKG (kinase cascades) and small GTPases Rac and Rho (cytoskeletal dynamics), as well as Src family kinases (adhesion and survival signals). Markers such as PCNA and p21 indicate effects on proliferation and cell-cycle arrest. Collectively, these demonstrate that non-neuronal cholinergic signalling is a versatile regulator of cellular phenotype, integrating environmental cues to control survival, growth, migration and functional specialisation. This underscores its relevance in both normal physiology and pathological contexts, including non-small-cell lung cancer [8,44,65,66].

A substantial body of research has indicated that the role of ACh in the immune system highlights its important function in modulating immune responses, which extends beyond its conventional role as a neurotransmitter [44,45]. Vagal afferent fibre-mediated suppression of inflammation is initiated by the release of ACh from these fibres, which binds to the α7nAChR on macrophages and other immune cells [67]. The presence of cholinergic components, including muscarinic (mAChRs) and nicotinic (nAChRs) receptors, in T cells, B cells and macrophages highlights the importance of ACh in regulating calcium signalling pathways, which are essential for innate and adaptive immunity. Notably, immune cells themselves can synthesise and release ACh, establishing an autocrine and paracrine communication system that amplifies their regulatory potential [68].

Importantly, ACh has been shown to support the functionality of T cells, B cells and macrophages. This contributes to our comprehensive understanding of immune mechanisms and potential therapeutic interventions, as demonstrated by Fujii et al. [68]. Specifically, the non-neuronal ACh expressed in lymphocytes plays a pivotal role in regulating immune function. Through their intrinsic cholinergic activity, lymphocytes contribute to processes such as T cell selection, maturation, and local vascular modulation, which occur independently of traditional cholinergic nerves [45]. Recent evidence suggests that ACh synthesised by T cells, alongside the endogenous α7 nAChR ligand SLURP-1, modulates cytokine production and promotes T cell development. This occurs by acting on α7 subunit of nicotinic acetylcholine receptors expressed on immune cells, thereby contributing to immune regulation [69]. These mechanisms highlight the integration of the cholinergic system into immune regulation and emphasise its importance in maintaining immune homeostasis. This dual regulation—via both neuronal and non-neuronal ACh—highlights the existence of a ‘non-neuronal cholinergic system’ as a distinct yet complementary component of the immune–neural interface [67,70].

Furthermore, it has been demonstrated that peptide ligands such as SLURP-1 and HCNP influence cholinergic activity in immune cells [71]. This creates a foundation for innovative immunomodulation research. As demonstrated by Mashimo et al. [44], the multifaceted role of ACh could be pivotal in developing novel therapeutic strategies for immune-related diseases. Binding results in the inhibition of the synthesis and release of pro-inflammatory cytokines, including TNF-α, IL-1β and IL-6. This mechanism functions independently of glucocorticoids and represents a rapid, localised anti-inflammatory response. Consequently, dysregulation of the CAP is now recognised as a hallmark of the pathophysiology of a range of disorders, from autoimmune diseases to metabolic syndrome [38,72]. Dysregulation of the CAP has been implicated in numerous chronic inflammatory diseases, including rheumatoid arthritis, inflammatory bowel disease (IBD), sepsis, and long-term effects of SARS-CoV-2 infection [44].

Recent evidence has transformed our understanding of the immune system by demonstrating that immune cells possess a functional non-neuronal cholinergic system. In this system, ACh-producing lymphocytes (particularly CD4+ T cells that express choline acetyltransferase, or ChAT+) synthesise and release ACh. This ACh then acts via muscarinic and nicotinic receptors, especially α7 nAChRs, on various immune cells. This modulates inflammation, cytokine production and antimicrobial defence mechanisms [44,73,74]. ChAT-positive lymphocytes have been demonstrated to be pivotal mediators of the “inflammatory reflex”, a neuroimmune communication pathway linking efferent vagal nerve activity to immune modulation in the spleen and intestine [75]. Importantly, ChAT+ T cells act as messengers, transmitting neural signals to splenic macrophages. This process has been shown to suppress TNF-α production via α7 nAChR activation, thereby contributing to the attenuation of systemic inflammation [76]. Mice deficient in these T cells exhibit impaired vasodilation, elevated blood pressure, reduced viral control and altered gut microbiota, highlighting their importance in maintaining vascular and immune balance [77,78]. These findings suggest that cholinergic lymphocytes serve as a crucial cellular link between neural inputs and peripheral immune effectors [46].

Furthermore, within the intestinal immune system, ChAT+ T cells, which are predominantly of the Th17 phenotype, have been shown to co-express IL-17A and IL-22 [79]. These cells contribute to the regulation of antimicrobial peptide (AMP) secretion and microbial composition. Deletion of ChAT in CD4+ T cells has been shown to result in decreased AMP expression, including lysozyme, defensin A and angiogenin-4. This leads to increased bacterial diversity within the gut lumen [79]. This highlights the dual role of ACh-producing lymphocytes in mucosal defence and maintaining host–microbiota symbiosis.

Research has demonstrated that the functional presence of cholinergic machinery—including ACh, ChAT, AChE, and multiple receptor subtypes (M1-M5 mAChRs and α7, α2, α5, and α9 nAChRs)—in T and B cells, macrophages, and dendritic cells supports the view of a broadly distributed, cell-intrinsic cholinergic regulatory network [68,73]. Indeed, activation of these receptors has been shown to enhance or suppress immune responses depending on the receptor subtype and cell type involved. For example, M1/M5 mAChR signalling promotes pro-inflammatory cytokine production, whereas activation of the α7 nAChR limits inflammation and antibody overproduction [68,73,80]. This receptor-specific divergence of cholinergic signalling underscores the complexity and plasticity of immune regulation by ACh.

Thus, the significance of these findings lies in the therapeutic potential of targeting the immune cholinergic system. Modulating ACh signalling using specific receptor agonists or antagonists could lead to new treatments for chronic inflammatory diseases, autoimmune conditions and even cancer [68,73]. Furthermore, understanding how immune cells participate in neural reflex arcs, particularly in the absence of direct vagal innervation—as in the spleen—could lead to more sophisticated models of neuroimmune integration and the development of bioelectronic medicine. Consequently, the immune cholinergic system is increasingly recognised as a fundamental regulator of immune homeostasis and a promising target for next-generation therapeutic strategies [72].

## 3. Mechanisms Linking Parasympathetic Function to Stress Regulation

### 3.1. Vagal Anti-Inflammatory Pathways and Modulation of the HPA Axis

**Figure 2:**
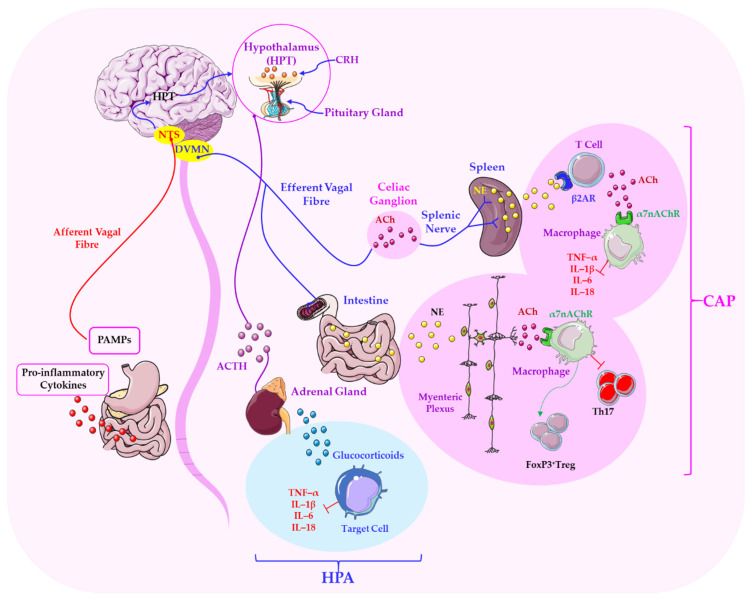
**The vagal cholinergic anti-inflammatory pathway and neuroendocrine regulation of the HPA axis.** The afferent fibres of the vagus nerve detect proinflammatory cytokines (e.g., TNF-α and IL-1β) and pathogen-associated molecular patterns in peripheral tissues. This information is transmitted to the nucleus of the solitary tract (NTS) in the brainstem, where it is integrated and efferent (motor) fibres of the vagus nerve are activated. These efferent endings then release acetylcholine (ACh) into inflamed tissues. This binds to α7 nicotinic acetylcholine receptors located on macrophages and other immune cells. Activation of these receptors inhibits the transcription of proinflammatory cytokine genes (such as *TNF-α*, *IL-1β* and *IL-6*), partly by suppressing the transcription factor NF-κB. The vagus nerve also modulates neuroendocrine stress responses by influencing the hypothalamic–pituitary–adrenal axis. Vagal afferent input to the NTS can regulate the activity of the paraventricular nucleus of the hypothalamus, either suppressing or enhancing its output. This, in turn, modulates the secretion of corticotropin-releasing hormone (CRH), which stimulates the anterior pituitary gland to secrete adrenocorticotropic hormone (ACTH). Ultimately, this leads to the synthesis and release of cortisol from the adrenal cortex. **Abbreviations:** Ach—acetylcholine; ACTH—adrenocorticotropic hormone; α7nAChRs—α7 nicotinic acetylcholine receptors; β2AR—beta-2 adrenergic receptor; CAP—cholinergic anti-inflammatory pathway; CRH—corticotropin-releasing hormone; DVMN—dorsal motor nucleus of the vagus; FoxP3^+^Treg—FoxP3^+^ regulatory T cells (Tregs)—subset of T lymphocytes characterized by the expression of the transcription factor FoxP3; HPA—hypothalamic–pituitary–adrenal axis; HPT—hypothalamus; IL-1β–interleukin 1β; IL-6—interleukin 6; IL-18—interleukin 18; NTS—nucleus of the solitary tract; NE—noradrenaline; PAMPs—pathogen-associated molecular patterns; Th17—Th17 cells—subset of CD4+ T; TNF-α—tumor necrosis factor-alpha. This figure was created using Servier Medical Art (available at https://smart.servier.com/) (accessed on 1 May 2025).

The dependency in question relates to interaction with the HPA axis and stress responsiveness. This is because the vagus nerve modulates neuroendocrine stress responses by influencing the HPA axis [81]. Tsigos and Chrousos [82] emphasised the pivotal function of the vagus nerve in regulating immune responses by integrating with the HPA axis and the autonomic nervous system. The vagus nerve transmits stress signals and regulates inflammation by activating neuroendocrine mechanisms, including glucocorticoids and catecholamines, which directly modulate immune function and suppress inflammation. This cholinergic anti-inflammatory pathway, which is mediated through nicotinic receptors on immune cells, demonstrates the influence of the nervous system on immune homeostasis. Importantly, dysregulation of this pathway has been linked to chronic inflammatory diseases and stress-related disorders [47,72], highlighting the clinical relevance of vagus–HPA interactions. These findings emphasise the significance of the vagus nerve in alleviating chronic, stress-induced pathological inflammation and in maintaining physiological equilibrium. Figure 2 shows the important interactions between the vagal cholinergic system and the HPA axis.

**Figure 3:**
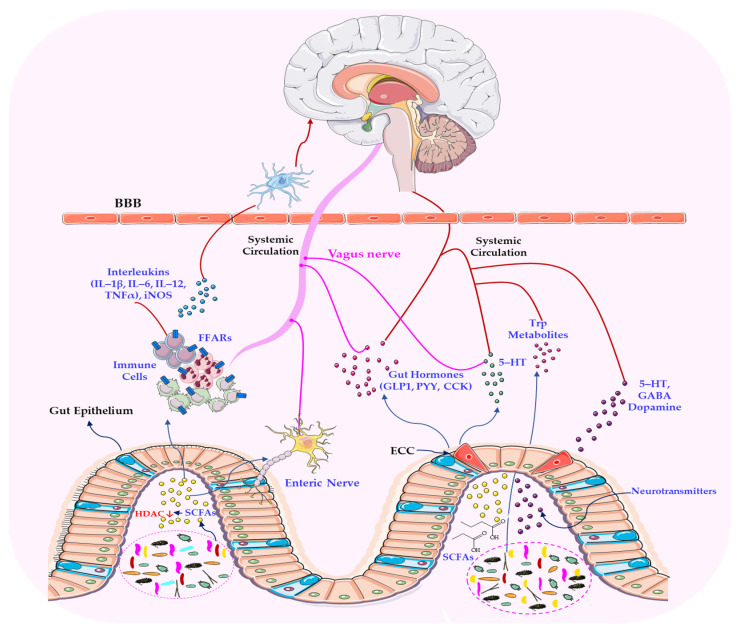
**Gut-to-brain signalling via the vagus nerve.** Afferent signals from the gut to the brain are transmitted via the vagus nerve, with significant contributions from enteroendocrine cells (EECs), epithelial cells and immune cells. These cells sense nutrients and microbial products and release hormones and neuropeptides, such as cholecystokinin (CCK), glucagon-like peptide-1 (GLP-1), peptide YY (PYY) and serotonin. These hormones activate vagal afferents. During inflammation, immune cells secrete cytokines such as IL-1β and TNF-α, which further influence vagal signalling. Gut microbiota modulate vagal activity by producing short-chain fatty acids, such as butyrate and propionate, which interact with free fatty acid receptors on intestinal cells. Butyrate plays a particularly important role in maintaining the integrity of the intestinal epithelial barrier by regulating gene expression through the inhibition of histone deacetylase and modulating inflammatory responses. In addition to SCFAs, gut microbes synthesise neuroactive compounds including GABA, serotonin, dopamine and tryptophan metabolites such as indole derivatives and kynurenine. These compounds can act directly on vagal afferents or indirectly via enteroendocrine and immune cell signalling, thereby contributing to neurochemical and behavioural modulation via the microbiota–gut–brain axis. **Abbreviations:** BBB—blood–brain barrier; CCK—cholecystokinin; GABA—γ-aminobutyric acid; GLP1—glucagon-like peptide-1; ECC—enterochromaffin cells; FFARs—free fatty acid receptors; HDAC—histone deacetylase; 5-HT—serotonin; iNOS—inducible nitric oxide synthase; IL-1β—interleukin 1β; IL-6—interleukin 6; IL-12—interleukin 12; PYY—peptide YY; SCRAs—short-chain fatty acids; TNF-α—tumor necrosis factor-alpha; Trp—tryptophan. This figure was created using Servier Medical Art (available at https://smart.servier.com/) (accessed on 1 May 2025).

Vagal afferents that project to the nucleus tractus solitarius (NTS) modulate the activity of the paraventricular nucleus (PVN) and corticotropin-releasing hormone (CRH) secretion, thereby regulating adrenocorticotropic hormone (ACTH) release and cortisol synthesis. Vagal tone dampens the excessive activation of the HPA axis and its associated metabolic, neurodegenerative, and immunosuppressive consequences [83]. Reduced vagal tone, often indicated by decreased heart rate variability (HRV), has been associated with increased stress responsiveness and conditions such as anxiety, depression and PTSD. This suggests that stress responses are influenced by vagal activity and that it can serve as an indicator of resilience and vulnerability [84]. Recent studies have also emphasised the importance of molecular communication with the gut microbiota in determining these dependencies. A growing body of research highlights the pivotal role of the vagus nerve in mediating gut–brain communication, particularly through its sensitivity to microbial metabolites [85,86,87]. It can therefore be hypothesised that the vagus nerve, as the primary component of the PSNS, plays a pivotal role in modulating stress responses by influencing the brain–gut axis. As shown in Figure 3, gut-to-brain signalling occurs via the vagus nerve. Afferent inputs from enteroendocrine, epithelial and immune cells form a bidirectional network in which neural, endocrine and immune pathways converge, linking emotional states with gastrointestinal and metabolic health.

As Breit et al. [40] emphasised, vagus nerve stimulation has been shown to enhance vagal tone. This inhibits the production of pro-inflammatory cytokines and impacts the monoaminergic brain systems that are critical for mood and anxiety disorders. Increased vagal tone has been shown to be strongly associated with improved stress regulation. Practices such as meditation and yoga, which enhance vagal activity, have been shown to promote resilience and alleviate anxiety and depression symptoms. Furthermore, the favourable effects of gut microbiota on mood and anxiety, which are partly mediated by the vagus nerve, highlight the interconnected nature of the brain–gut axis in stress adaptation mechanisms [40].

Short-chain fatty acids (SCFAs), such as butyrate and propionate, which are produced by the fermentation of dietary fibres by microbes, have been shown to modulate vagal afferent activity by interacting with free fatty acid receptors (FFARs) and influencing enteroendocrine signalling [88,89]. Additionally, microbial-derived gamma-aminobutyric acid (GABA), serotonin (5-HT) and tryptophan metabolites (e.g., kynurenine and indole derivatives) can modulate vagal signalling directly or via epithelial and immune cells [90]. For example, serotonin produced by enterochromaffin cells activates vagal terminals that express 5-HT3 receptors. This influences gastrointestinal motility, mood and cognitive function [91]. This neurochemical diversity illustrates the complex interplay between the microbiota, the vagal afferents and the central neural circuits involved in stress regulation. Disruptions to the composition of the microbiota (dysbiosis) have been shown to interfere with this communication, thereby contributing to the pathophysiology of functional gastrointestinal disorders, neurodevelopmental conditions (e.g., autism spectrum disorder) and mood disorders (e.g., depression) [92,93].

In conclusion, ACh-producing lymphocytes are central regulators of both cytokine production and the immune response to microbes. They also serve as a bridge between the nervous and immune systems, representing a critical axis for maintaining immune equilibrium and offering promising targets for future immunomodulatory therapies. Together with vagal–HPA interactions and gut–brain communication, these mechanisms highlight an integrated framework through which the parasympathetic system governs stress adaptation and systemic homeostasis [46].

### 3.2. Parasympathetic Influence on Metabolic Flexibility and Stress Adaptation

It is important to note that the PSNS, particularly via the vagus nerve, acts as a key counter-regulatory mechanism to the HPA axis [94]. While the sympathetic nervous system orchestrates the acute stress response, characterised by heightened alertness and the mobilisation of energy resources, the PSNS promotes restorative and homeostatic processes, including metabolic recovery, anti-inflammatory signalling and emotional regulation [60]. Vagal tone, which reflects parasympathetic activity, has been shown to be inversely correlated with cortisol levels and markers of psychological stress. Higher vagal tone has been associated with greater emotional resilience and faster recovery from stress, particularly in situations that emphasise the importance of gastrointestinal vagus nerve signalling in regulating neurocognitive processes that underpin various adaptive behavioural responses [95,96]. Moreover, reduced vagal tone, which is often seen in people with metabolic syndrome and obesity, has been linked to impaired metabolic flexibility and decreased resilience to stress [97]. This further highlights the important role of parasympathetic regulation in mental and physical health [98].

Previous studies have demonstrated that intracellular metabolic transformation pathways are significantly modified by exogenous ACh in a rat model [99]. This neurotransmitter’s effect is associated with an increase in intracellular calcium (Ca^2+^) concentration resulting from either its influx from the extracellular space or its release from intracellular stores, primarily the endoplasmic reticulum [100]. ACh binding to cholinergic receptors has also been shown to increase mitochondrial Ca^2+^ concentration. This mitochondrial calcium transport is activated by hormones such as glucagon and adrenaline, and is linked to their ability to promote succinate (SC) oxidation and subsequent proton gradient generation [49,99]. Under the influence of adrenaline, the mitochondrial calcium buffering capacity—namely, the maximal amount of Ca^2+^ that the mitochondria can uptake without compromising their functional integrity—increases [101].

**Figure 4:**
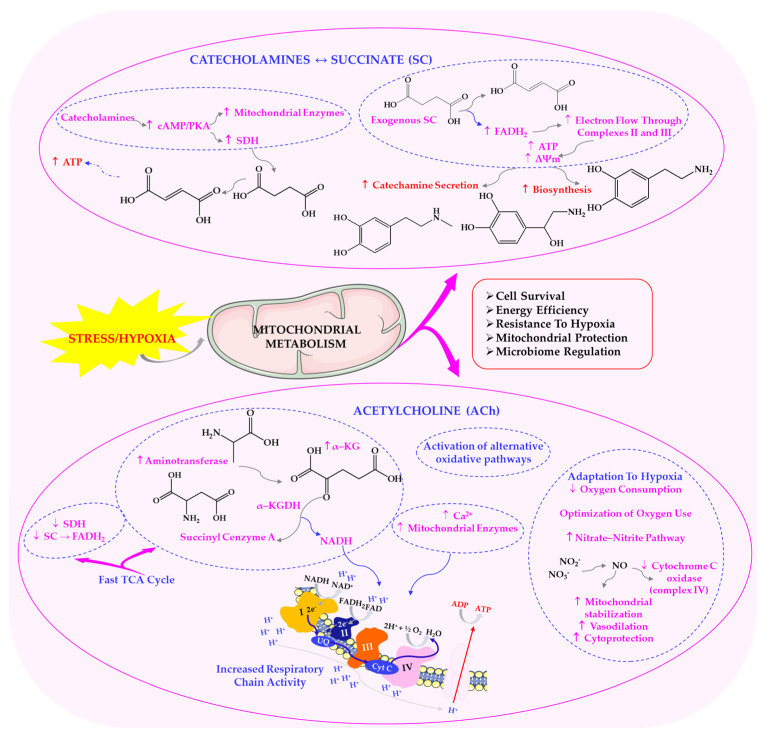
**Cholinergic and metabolic regulation of mitochondrial adaptation under hypoxic stress.** Under hypoxic stress, a bidirectional relationship emerges between energy metabolism, hormonal signalling and receptor activity. Succinate (SC) oxidation is modulated by catecholamines, while the presence of exogenous SC increases the turnover of catecholamines, indicating that SC plays a regulatory role in synaptic transmission. Acetylcholine activates α-ketoglutarate oxidation via stimulation of aminotransferase and inhibits succinate dehydrogenase. This supports rapid flux in the TCA cycle independent of oxidative phosphorylation. Hypoxia also induces a metabolic shift towards nitrate/nitrite respiration, enhancing survival. Therefore, cholinergic receptor function is essential for mitochondrial and cellular adaptation to oxygen deficiency, partly mediated by increased nitric oxide levels. **Abbreviations:** ACh—acetylcholine; ADP—adenosine diphosphate; ATP—adenosine triphosphate; cAMP—cyclic adenosine monophosphate; Cyc C—cytochrome C; α-KG—α-ketoglutarate; α-KGDH—α-ketoglutarate dehydrogenase; FADH_2_—flavin adenine dinucleotide; NADH—nicotinamide adenine dinucleotide; NO—nitric oxide; PKA—protein kinase A; SC—succinate; SDH—succinate dehydrogenase; TCA—tricarboxylic acid cycle; UQ—ubiquinone; ΔΨm—mitochondrial membrane potential. This figure was created using Servier Medical Art (available at https://smart.servier.com/) (accessed on 1 May 2025).

As the primary neurotransmitter of the PSNS, ACh affects cellular metabolism by stimulating mitochondrial enzymes and enhancing bioenergetic processes, as demonstrated in various animal models, including zebrafish [102]. These findings highlight that, in addition to regulating immune and neuroendocrine responses, cholinergic signalling directly influences mitochondrial efficiency, redox balance and energy allocation. These processes are essential for adapting to stressful conditions [49,99]. As shown in Figure 4, a bidirectional relationship arises between energy metabolism, hormonal signalling and receptor activity under hypoxic stress. This highlights the cholinergic and metabolic mechanisms that govern mitochondrial adaptation.

It has been demonstrated that there is a dynamic interaction between energy metabolism, hormonal signalling and cellular receptor systems, particularly under hypoxic conditions [103,104]. This is consistent with the hypothesis of a functional feedback loop, whereby SC oxidation is regulated by catecholamines and, in turn, exogenous SC stimulates catecholamine metabolism [100,103,104]. This bidirectional relationship suggests that succinate plays a direct role in regulating synaptic transmission. Furthermore, ACh can activate the oxidation of α-ketoglutarate in mitochondria by stimulating aminotransferase reactions while simultaneously inhibiting succinate dehydrogenase (SDH) activity. This mechanism supports the concept of a ‘fast cycle’ within the Krebs cycle, which is activated under various types of functional load (e.g., hypoxia, stress, adaptation) and aligns with findings on α-ketoglutarate’s cholinomimetic properties [100,105]. Importantly, these interactions highlight the convergence of neurotransmitter signalling and intermediary metabolism, providing an adaptive advantage under conditions of limited oxygen supply.

Previous studies have shown that ACh activates oxidative pathways that are not directly linked to oxidative phosphorylation processes. Under hypoxic conditions, for instance, shifting cellular respiration towards alternative nitrate–nitrite respiration enhances the survival of animals exposed to acute hypoxia (7% O_2_) [103,104]. Therefore, the functional state of cholinergic receptors is critical for cellular and mitochondrial metabolic remodelling involving not only ACh but also nitric oxide (NO). The concentration of NO rises significantly during adaptation to oxygen deficiency [100,103,104]. The synergistic interaction between ACh and NO ensures the preservation of redox balance, vascular tone and mitochondrial efficiency, all of which are crucial for adaptation to hypoxic stress.

It has been shown that ACh optimises oxygen uptake and utilisation under extreme hypoxic stress, and that parasympathetic regulation modulates individual resistance in animals. Based on these findings, Kurhaluk et al. (2024) have proposed a comprehensive framework to explain how mitochondrial and cellular function is regulated [103,104]. This framework encompasses high-energy compounds, hormones, metabolic cofactors and second messengers. The coordinated activity of these regulatory networks is essential for sustaining oxygen consumption and maximising the efficiency of ATP production processes in mitochondria, in accordance with the cell’s functional state [106,107]. Such integrative regulation demonstrates how parasympathetic inputs can fine-tune metabolic plasticity to ensure both immediate survival and long-term stress resilience [108].

Wessler et al. (1999, 2001) proposed the concept of acetylcholine as a ‘cholinergic pitfall’, referring to its ubiquitous, evolutionarily conserved and multifunctional nature, which makes it difficult to intervene in cholinergic signalling pathways in a straightforward and targeted way [109,110]. ACh is a universal cellular molecule present in all biological systems, including the human body, and it functions as a prominent neurotransmitter that is widely expressed in both neuronal and non-neuronal cells, as well as in both prokaryotic and eukaryotic organisms. This suggests that ACh originated early in evolution. In humans, ACh and its synthesising enzyme, choline acetyltransferase, are present in epithelial, mesothelial, endothelial and muscle cells, as well as immune cells such as leukocytes and microglia, which actively produce ACh [109,110]. This highlights the critical role of ACh in neuroimmune regulation.

The non-neuronal functions of ACh are regulated by cholinesterase activity and are mediated via nicotinic and muscarinic receptors [7,44,45,65,66,67,68,73]. These receptors influence key cellular processes, including mitosis, differentiation, cytoskeletal organisation, secretion, absorption and immune responses. Collectively termed ‘trophic properties’, these roles underscore the necessity for further investigation into ACh’s cellular effects and its involvement in chronic inflammatory diseases, with the aim of elucidating new therapeutic strategies [109,110]. Of particular note, ACh’s dual role as both a neurotransmitter and a metabolic modulator suggests that its signalling dysregulation may contribute to the onset of systemic pathologies, including neurodegeneration and metabolic disorders.

Further evidence supports the role of ACh as an endogenous vasoactive agent that stimulates NO production, highlighting its contribution to adaptive responses under hypoxic and other extreme conditions [103,104]. Physiological variability in the hypoxic stress response is reflected in mitochondrial respiration: animals with high or low resistance to oxygen deficiency exhibit different profiles of activity in the alanine/aspartate aminotransferase and succinate dehydrogenase pathways, as well as different coupling efficiencies between respiration and ATP synthesis. Despite reduced mitochondrial phosphorylation efficiency—indicating a lower amount of ATP produced per unit of oxygen consumed—high-resistance animals maintain strong coupling between respiration and ATP production. This suggests more effective oxygen utilisation and energy conversion. This interindividual variability may depend, at least in part, on vagal modulation of mitochondrial dynamics and microbiota-derived signals, which together shape systemic stress responses [111].

Therefore, the existing literature emphasises the pivotal role of ACh in regulating cellular and mitochondrial metabolism, particularly in conditions of oxidative stress and hypoxia [112,113]. ACh modulates intracellular signalling pathways involving calcium, cyclic nucleotides and oxidative phosphorylation. This influences not only localised bioenergetic processes, but also the systemic adaptive response. The interactions observed between neurotransmission, mitochondrial enzymatic activity and hormonal signalling emphasise the existence of a complex, reciprocal regulatory network that ensures metabolic flexibility in response to environmental challenges [103,104,109,110]. Collectively, these insights reinforce the idea of ACh as a key integrative mediator at the intersection of neural, immune, and metabolic regulation, offering a promising direction for future bioelectronic and pharmacological interventions.

## 4. Strategic Modulation of Parasympathetic Activity

### 4.1. Lifestyle-Based Interventions: Intermittent Hypoxia, Exercise and Diet

Although there is strong preclinical evidence supporting the therapeutic potential of intermittent hypoxia, clinical validation through large-scale randomised controlled trials is limited. Therefore, current conclusions should be interpreted with caution until standardised therapeutic protocols and safety parameters have been established. Intermittent hypoxia training (IHT) has been mechanistically linked to the regulation of the parasympathetic nervous system due to its multifaceted impact on mitochondrial function, nitric oxide dynamics, and the modulation of oxidative stress [114,115]. It is important to understand the difference between mild, controlled IHT, which can have beneficial physiological and stress-modulating effects, and chronic or severe intermittent hypoxia, which can have detrimental consequences [49,106,108].

Experimental data from animal models of acute hypoxia (AH; exposure to 7% O_2_ in N_2_ for 30 min) showed that intermittent hypoxia training (IHT; exposure to 11% O_2_ in N_2_ for 15-min sessions with 15 min rest intervals, five times daily for 14 days) prevented oxidative stress activation, reduced lipid peroxidation, and limited oxidative protein damage. Under AH conditions, an increase in epinephrine content was accompanied by a shift in mitochondrial substrate utilisation towards SC oxidation and decreased α-ketoglutarate utilisation [104]. Following the IHT course, SC-dependent mitochondrial oxidation normalised, nitrite anion levels increased, and energy metabolism improved [103,104]. These findings suggest that controlled mild hypoxia induces adaptive mechanisms that enhance metabolic and oxidative resilience. However, these findings require validation in controlled clinical settings to confirm their clinical relevance.

The increased NO bioavailability observed following IHT enhances vasodilation and tissue oxygenation, and plays a central role in promoting vagal tone, a key determinant of parasympathetic activity [100,103,104]. Increased parasympathetic outflow results in a slower heart rate, improved baroreflex sensitivity, and reduced systemic inflammation. Furthermore, IHT-induced activation of hypoxia-inducible factors (HIFs) and modulation of oxidative phosphorylation pathways enhance cellular resilience and neuroprotection, which are processes inherently supported by parasympathetic dominance. Therefore, adapting to intermittent hypoxia, particularly through L-arginine supplementation to boost endogenous NO levels, could encourage a shift towards parasympathetic regulation, offering cardiovascular, metabolic and cognitive health benefits [49,100,104]. These molecular dependencies are illustrated in Figure 4.

In addition, IHT appears to significantly modulate the gut microbiota via NO-mediated signalling and systemic redox balance. Recent research highlights nitric oxide synthase (NOS) as a key mediator in maintaining gut microbiota homeostasis via immune-related signalling pathways in aquatic species; both invertebrate and vertebrate. Hong et al. demonstrated in shrimp (*Marsupenaeus japonicus*) that an oral infection with *Vibrio anguillarum* induces NOS expression and NO production, activating the ERK-NF-κB-like (dorsal) pathway and promoting the expression of antimicrobial peptides (AMPs) and effective bacterial clearance [20]. RNA interference of NOS or chemical inhibition via L-NMMA resulted in significantly reduced NO levels and an increased bacterial burden in the gut. However, NO supplementation reversed these effects, highlighting NO as a central signalling molecule that bridges parasympathetic activity, immune regulation and gut microbiota homeostasis [20]. Further research using genetically engineered zebrafish revealed that knocking out the inducible form of NOS (iNOS or NOS2) significantly altered the diversity and composition of gut microbes. Huang et al. reported a significant decrease in *Vibrio* and an increase in *Aeromonas* spp. in iNOS-deficient zebrafish, particularly in double knockout lines [21]. Although there was minimal impact on intestinal tissue integrity, transcriptomic analysis revealed substantial changes in immune and metabolic pathways, including complement and peroxisome proliferator-activated receptor (PPAR) signalling. This implicates iNOS as a key modulator of gut microbial ecology and host immunometabolism [21].

Regular physical exercise is a powerful modulator of parasympathetic activity, activating a complex network of metabolic and signalling pathways that extend from skeletal muscle to central autonomic circuits [116]. During sustained muscular activity, the activation of AMP-activated protein kinase and peroxisome proliferator-activated receptor gamma coactivator 1-alpha (PGC-1α) stimulates mitochondrial biogenesis and enhances oxidative phosphorylation. This process also reshapes the cellular redox environment by limiting the accumulation of reactive oxygen species, thereby reducing oxidative injury in peripheral and central tissues. These biochemical adjustments reverberate through vagal afferent pathways, improving baroreflex sensitivity and restoring autonomic balance [117]. Meanwhile, exercise-induced myokines, including irisin and brain-derived neurotrophic factor, penetrate the blood–brain barrier and facilitate plasticity in brainstem nuclei that control parasympathetic output. This process strengthens vagal tone and dampens chronic sympathetic hyperactivity, which is associated with cardiometabolic disorders [118]. These effects demonstrate how physical activity can reinforce parasympathetic dominance and enhance systemic resilience to stress and metabolic disturbances.

Dietary interventions characterised by caloric moderation and the presence of bioactive compounds, such as omega-3 fatty acids and polyphenols, can complement the autonomic benefits of exercise [119]. This is achieved by influencing nutrient-sensing and stress-responsive biochemical pathways that determine cellular resilience. Restricting energy intake or strategically modulating the composition of macronutrients enhances sirtuin activity and promotes nicotinamide adenine dinucleotide (NAD^+^) availability. Together, these processes counteract mitochondrial dysfunction and prolong cellular healthspan. Meanwhile, suppressing NF-κB-mediated inflammatory cascades and activating Nrf2-dependent antioxidant defences reduces systemic inflammation and stabilises neuronal excitability within the dorsal motor nucleus of the vagus [120]. In this way, diet synergistically supports parasympathetic activity and the cholinergic anti-inflammatory reflex, integrating metabolic, immune, and neural pathways.

Taken together, these studies provide compelling evidence that NOS activity regulates host–microbe interactions via conserved molecular mechanisms, particularly the ERK-NF-κB axis, as well as through the activation of Nrf2-dependent antioxidant defences and immunometabolic modulation. Therefore, lifestyle-based interventions, including intermittent hypoxia, exercise and diet, converge mechanistically on parasympathetic regulation, metabolic flexibility and immune homeostasis, highlighting their potential in preventive and therapeutic strategies for human health.

### 4.2. Novel Approaches to Enhancing Vagal Tone and Physiological Resilience

Reduced chronic low-grade inflammation and lipoperoxidation in peripheral tissues suggests improved gut barrier function and a potential shift in microbial composition towards anti-inflammatory, butyrate-producing bacteria. Although reactive oxygen species (ROS) are elevated during IHT, they may act as signalling molecules in controlled doses to promote mitophagy and maintain epithelial homeostasis in the gut. At the same time, elevated levels of circulating NO and nitrite may influence microbial metabolism, favouring nitrate-reducing commensals and altering luminal redox potential [99]. Due to the bidirectional communication of the gut–brain axis, which includes vagus-mediated pathways and microbial metabolite signalling via SCFAs, the systemic adaptations induced by IHT may provide feedback to support neuroimmune balance and parasympathetic tone. This highlights a potential synergistic link between mitochondrial adaptation, gut microbiota homeostasis, and autonomic regulation [103,104].

Recent research has begun to elucidate the intricate connections between IHT and the gut microbiota, revealing significant implications for metabolism, inflammation and cognitive function. For example, Van Meijel et al. (2022) demonstrated that exposure to mild intermittent hypoxia can significantly alter the composition of the gut microbiota in overweight and obese men [121]. They found that the abundance of microbial taxa associated with metabolic health increased, suggesting that even low-dose hypoxic exposures can shift microbial communities towards an anti-inflammatory and metabolically favourable profile. A companion study [122] further supports the hypothesis that low-dose hypoxic exposures can induce these changes, showing that IHT caused proteomic remodelling in subcutaneous adipose tissue, favouring pathways linked to lipid metabolism and mitochondrial function. These systemic effects are likely mediated, at least in part, by microbiota-derived signals that interface with host neuroimmune and metabolic networks.

Evidence from both animal and human models further substantiates the concept that gut microbiota play a pivotal mediating role in hypoxia-induced systemic effects. For example, Moreno-Indias et al. (2016) [123] investigated whether changes in the gut microbiota and circulating endotoxemia induced by intermittent hypoxia (IH) in mice, which mimics obstructive sleep apnoea, are reversible after a period of normoxia. While bacterial richness and diversity appeared similar following recovery, IH-exposed mice exhibited an altered composition of gut microbiota (increased *Firmicutes* and *Deferribacteres*, and decreased *Bacteroidetes*), as well as elevated plasma lipopolysaccharide (LPS) levels [123]. This establishes a connection between hypoxic stress and gut dysbiosis. Similarly, Guo et al. (2024) found that adult patients with obstructive sleep apnoea—a clinical condition characterised by chronic intermittent hypoxia—exhibited microbiota alterations associated with increased inflammatory burden and metabolic dysfunction [124].

Notably, while chronic or severe hypoxia often leads to dysbiosis and systemic inflammation, controlled mild intermittent hypoxia has been found to have protective effects, potentially via hormetic mechanisms. In a seminal study, Olson et al. (2021) demonstrated that hypoxia-induced microbial changes can modulate host cognition and behaviour, implicating the gut–brain axis in hypoxia-mediated outcomes [125]. They found that specific microbial shifts under hypoxic conditions may influence neurocognitive function via metabolite signalling and immune modulation. This is consistent with the findings of Zhou et al. (2021), who demonstrated that the gut microbiota modulate cardiac transcriptional responses to intermittent hypoxia, thereby emphasising the systemic reach of microbiome-mediated hypoxic adaptations [126]. These studies collectively support the notion that mild IHT functions as a hormetic stressor that optimises host physiology via gut–brain–immune interactions.

Further studies have clarified the dose-dependent nature of interactions between IHT and the microbiota. Dong et al. (2024) demonstrated that controlled reoxygenation following intermittent hypoxia mitigates gut dysbiosis and systemic inflammation in obese rats, thereby emphasising the importance of cyclic oxygen dynamics [127]. Meanwhile, Li and Shi (2024) observed significant changes in the microbiota and metabolome of rats exposed to chronic intermittent hypoxia, revealing a connection between dysbiosis and altered lipid and bile acid profiles that could contribute to metabolic syndrome progression [128]. Bodkin et al. (2022) reported that intermittent hypoxia in the early life stages alters the colonisation of gut microbes in neonatal rats [129]. This effect can be modified by antioxidant or fish oil supplementation, suggesting that dietary and pharmacological contexts may influence the efficacy of IHT in shaping the microbiome.

The findings of the present studies indicate that the effects of intermittent hypoxia on gut microbiota functionality as an antistress tool are highly context-dependent. They are shaped by factors such as exposure duration and intensity, host metabolic status, and concurrent lifestyle factors, including diet and physical activity [120]. Notably, when incorporated into a structured, health-promoting lifestyle, mild IHT can act as a hormetic stimulus, enhancing metabolic flexibility, supporting microbial diversity, and reducing systemic inflammation. This reinforces the notion previously discussed that, when combined with exercise, positive emotional states and parasympathetic activation, intermittent hypoxia can modulate the gut–brain–immune axis synergistically, promoting whole-body homeostasis and physiological resilience.

### 4.3. Integration with Microbiota-Targeted Strategies

**Figure 5:**
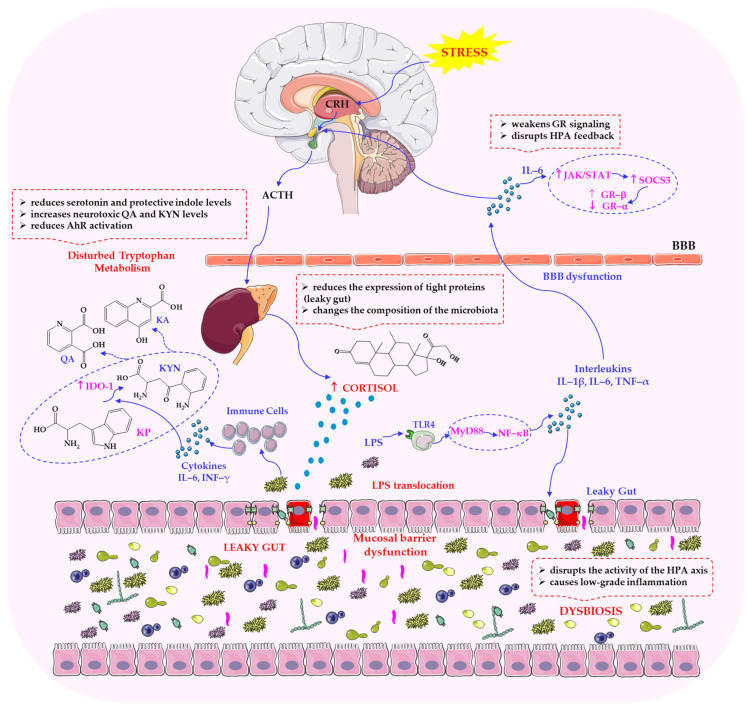
**Interaction between the HPA axis and the gut microbiota in stressful situations.** Prolonged cortisol secretion in response to chronic stress can disrupt the composition of the gut microbiota, leading to dysbiosis. This promotes the overgrowth of Gram-negative bacteria, which produce increased levels of LPS. LPS then compromises the integrity of the intestinal barrier by increasing epithelial permeability. Once weakened, LPS can translocate into the lamina propria, where it binds to TLR4 on the surfaces of intestinal dendritic cells, macrophages and epithelial cells. This interaction activates a signalling cascade involving adaptor proteins such as MyD88, ultimately leading to the activation of NF-κB. NF-κB then triggers the transcription of genes that encode proinflammatory cytokines, such as TNF-α, IL-6 and IL-1β. This results in chronic, low-grade inflammation. Elevated levels of IL-6 and other cytokines can impair the expression of glucocorticoid receptors through JAK/STAT signalling, thereby weakening negative feedback regulation of the HPA axis. This dysregulation perpetuates elevated cortisol levels during chronic stress. In the context of dysbiosis, cytokines such as IL-6 and IFN-γ stimulate IDO-1 activity, shifting tryptophan metabolism towards the kynurenine pathway. This increases the production of neurotoxic metabolites such as KYN and QA, while the levels of serotonin and protective indole derivatives decline. Consequently, the activation of AhR, which plays a role in maintaining mucosal immune homeostasis, is reduced. **Abbreviations:** ACTH—adrenocorticotropic hormone; AhR—aryl hydrocarbon receptor; BBB—blood–brain barrier; CRH—corticotropin-releasing hormone; GR-α/β—glucocorticoid receptors; HPA—hypothalamic–pituitary–adrenal axis; IDO-1—indoleamine 2,3-dioxygenase; IFN-γ—interferon-gamma; IL-1β—interleukin 1β; IL-6—interleukin 6; JAK/STAT—Janus kinases/signal transducer and activator of transcription; KA—kynurenic acid; KP—kynurenine pathway; KYN—kynurenine; LPS—lipopolysaccharides; MyD88—myeloid differentiation primary response 88; NF-κB—nuclear factor-kappa B; TLR4—Toll-like receptor 4; SOCS3—suppressor of cytokine signaling 3; TNF-α—tumor necrosis factor-alpha; QA—quinolinic acid. This figure was created using Servier Medical Art (available at https://smart.servier.com/) (accessed on 1 May 2025).

The brain–gut–microbiome axis is increasingly recognised as a key mediator of communication between the central nervous system and the gastrointestinal tract [130]. This system relies on a combination of neural pathways (particularly the vagus nerve), immune signalling, SCFAs, and the regulation of neurotransmitter availability. A growing body of research has demonstrated that gut microbiota can influence levels of serotonin, dopamine and GABA, which are vital for regulating emotional and cognitive responses to stress [131]. Disruptions in microbial composition, often termed dysbiosis, can impair the production of these neuroactive metabolites, thereby altering neuroendocrine responses and behavioural outcomes [132]. Figure 5 illustrates the complex, bidirectional interactions between the HPA axis and the gut microbiota under stressful conditions. It demonstrates the collaborative efforts of microbial metabolites, immune signalling and neuroendocrine factors in modulating stress responses and influencing the host’s physiological and behavioural adaptations.

In recent years, researchers have focused on the contribution of this axis to psychological and emotional regulation [133]. There is growing evidence that certain microbial strains or their metabolites can enhance resilience to stress by modulating vagal tone, regulating the HPA axis and influencing neuroinflammatory pathways. Conversely, others may contribute to anxiety- or depression-like behaviour. This has led to the exploration of microbiota-based interventions, such as targeted probiotics, prebiotics or faecal microbiota transplantation, as potential tools for modulating brain function via the gut [134]. These interventions are particularly promising when combined with lifestyle strategies that promote parasympathetic activation, such as intermittent hypoxia training, exercise and diet, as this creates a synergistic environment for neuroimmune regulation.

A salient aspect is the differential responsiveness of this axis to various stressors, particularly psychological and social ones [130]. For example, animal models, such as those involving maternal separation or social defeat, have consistently shown that stress can modify the composition of the microbiota in a way that reflects changes in behaviour and physiology [135]. In humans, individuals exposed to chronic social stress often exhibit signs of dysbiosis, highlighting a feedback loop in which emotional strain can further impair gut homeostasis, potentially exacerbating stress-related physiological and cognitive dysfunctions [136]. Many findings provide substantial evidence to support the hypothesis that the microbiome should be regarded as both a consequence and a causal driver of stress-related outcomes, emphasising its central role in integrated neuroimmune and metabolic regulation [10,137].

It has been demonstrated that the HPA axis plays a pivotal role in the body’s stress response system by orchestrating the secretion of stress hormones such as cortisol [138]. The initial research demonstrating this connection was conducted using animal models. Huo et al. (2017) observed that germ-free (GF) mice, characterised by an absence of gut microbiota, exhibited an exaggerated HPA axis response to mild stressors [139]. These mice had significantly higher levels of CRH, ACTH and corticosterone than specific-pathogen-free controls. This heightened stress response can be reversed by colonising GF mice with specific microbial strains. For example, Saturio et al. (2021) found that *Bifidobacterium infantis* normalises ACTH and corticosterone levels when given during early development [140]. This suggests that there is a critical period during which the microbiota can influence neuroendocrine reactivity. These findings highlight that microbial input during developmental windows affects not only immediate neuroendocrine function but also programmes long-term stress resilience.

A growing body of evidence emphasises the important role of the PSNS, particularly the vagus nerve, in mediating communication between the gut microbiota and distant organs such as the brain, liver, and endocrine pancreas. As summarised in Table 1, select preclinical and clinical studies have investigated how gut-derived signals, including microbial metabolites and specific bacterial taxa, affect physiological processes via parasympathetic pathways. Taken together, these studies demonstrate that parasympathetic activation or disruption impacts various host functions, including stress reactivity, immune signalling, metabolic regulation and cognitive and emotional behaviour. This emphasises the importance of considering the sympathetic-HPA and parasympathetic-vagal branches as integrated regulators of stress adaptation.

The extant literature clearly shows that several mechanisms have been proposed at the molecular level to explain how microbial signals influence the HPA axis [145]. One such mechanism involves SCFAs, particularly butyrate, produced by the bacterial fermentation of dietary fibre [146]. Butyrate acts as an inhibitor of histone deacetylases (HDACs), thereby modifying chromatin structure and enhancing the transcription of genes such as *Crh* in the paraventricular nucleus of the hypothalamus. This epigenetic regulation establishes a direct link between microbial metabolism and central neuroendocrine gene expression [147]. Beyond epigenetic regulation, SCFAs also serve as energy substrates for colonocytes and modulators of systemic metabolism, thereby further linking microbiota activity to host stress physiology [148].

Another mechanism of action focuses on the sensitivity of glucocorticoid receptors (GRs) in the brain [149]. Sorrells and Sapolsky (2007) highlighted this mechanism through which stress mediators influence neuroimmune interactions [150]. They demonstrated that glucocorticoids, which are traditionally viewed as anti-inflammatory, can have pro-inflammatory effects depending on the context. While acute glucocorticoid exposure in certain peripheral settings has been shown to facilitate immune activation, chronic exposure within the central nervous system promotes processes such as increased immune cell migration, amplified cytokine release and activation of transcription factors, including NF-κB [151]. Together, these findings redefine glucocorticoids as dual regulators of immune function, capable of dampening or enhancing inflammatory activity depending on timing, cellular targets and physiological state [152]. This has profound implications for our understanding of stress-related neuropathology and for the design of therapeutic strategies aimed at modulating glucocorticoid signalling.

Inflammatory cytokines, such as IL-6, whose levels increase in cases of dysbiosis, have been shown to impair GR function by activating the JAK/STAT3 pathway [153]. This results in decreased GR expression and nuclear translocation in hippocampal neurons, weakening negative feedback inhibition of the HPA axis and prolonging cortisol secretion in response to stress [150,154]. Thus, the interplay between pro-inflammatory cytokines and glucocorticoid sensitivity provides a molecular explanation for how gut dysbiosis may exacerbate maladaptive stress responses.

Evidence is also emerging that the vagus nerve plays a significant role in microbiota–HPA interactions [155]. Certain commensal bacteria have been observed to stimulate afferent fibres of the vagus nerve by activating Toll-like receptors (TLRs) and nucleotide-binding oligomerisation domain (NOD) receptors on intestinal epithelial and enteroendocrine cells. This sensory input then modulates hypothalamic activity. Indeed, vagotomy has been shown to eliminate the anti-anxiety and HPA-modulating effects of probiotic administration in animal models, which further confirms the critical role of the vagus nerve [156]. For example, SCFAs such as butyrate have been found to regulate the expression of glucocorticoid receptors in brain regions involved in HPA regulation, as demonstrated in rat models exhibiting autism-like behaviours following treatment with sodium butyrate [157]. There are also indications that microbiota-related signals affect the release of corticotropin-releasing hormone (CRH) in the hypothalamus, which activates the HPA cascade. This was demonstrated by Herman et al. [15]. Taken together, these mechanisms illustrate that the microbiota is an active regulator of neuroendocrine homeostasis, not a passive bystander.

This assertion takes on particular significance when considered in the context of chronic stress, which can be experienced through long-term caregiving, working in high-pressure environments, or exposure to ongoing trauma [154,158]. The HPA axis is known to play a role in numerous health problems, ranging from immune dysfunction to cognitive decline. Some findings suggest that the gut microbiota may either buffer or exacerbate these effects. For instance, increased microbial diversity and the presence of anti-inflammatory metabolites, such as butyrate and indole derivatives, have been linked to more adaptive HPA regulation and reduced allostatic load [10]. Therefore, it is hypothesised that promoting microbial diversity and metabolite production could restore adaptive HPA signalling and mitigate the long-term physiological effects of stress [158].

## 5. The Gut Microbiome as a Therapeutic Target in Stress

### 5.1. Microbiome-Mediated Regulation of Stress Responses

The vagus nerve integrates immune signals, microbial metabolites and neurotransmitter activity into a coherent communication channel. This serves as a physiological bridge, linking gut microbiome activity to stress-related brain circuits [10,143,159]. Microbial metabolites, such as SCFAs, GABA, serotonin, and indole derivatives, act on enteroendocrine and enterochromaffin cells within the intestinal epithelium. This prompts the release of signalling molecules, including 5-HT (serotonin), peptide YY (PYY), and glucagon-like peptide-1 (GLP-1), which bind to vagal afferent terminals in the mucosa (Figure 5) [160]. Activation of these afferents sends signals to the nucleus tractus solitarius in the brainstem, which projects to higher regions involved in stress regulation, including the hypothalamus and amygdala [161]. Microbially derived GABA and other neurotransmitter analogues can directly activate GABA receptors on vagal afferents, thereby modulating their excitability and influencing stress reactivity and emotional behaviour [162,163]. The vagus nerve also regulates intestinal inflammation via the cholinergic anti-inflammatory pathway through its efferent projections. ACh activates α7 nicotinic receptors on macrophages, suppressing pro-inflammatory cytokines such as TNF-α and IL-6 [164].

Saturio et al. [140], Tette et al. [163], Lei et al. [133], and Savulescu-Fiedler et al. [159] have convincingly demonstrated that the composition of the gut microbiome has a strong influence on the regulation of stress, modulating immune responses and the metabolism of neuroactive compounds. In contrast, Huo et al. (2017) [139] demonstrated that dysbiosis can result in the excessive activation of immunocompetent cells and the release of pro-inflammatory cytokines such as IL-6, TNF-α and IFN-γ. These cytokines activate intracellular cascades including MAPK kinases and NF-κB. This amplifies inflammation and affects neuronal activity, either directly or via activation of the HPA axis [40]. Beyond immune signalling, the gut microbiota regulates tryptophan metabolism, which is essential for serotonin production [165]. Under homeostatic conditions, tryptophan is processed via the serotonin pathway by TPH1/TPH2 and AADC to yield 5-HT, as demonstrated by Savelieva et al. [166]. Under chronic stress or immune activation, however, the kynurenine pathway predominates: IDO1/IDO2 and TDO convert tryptophan to N-formylkynurenine [167], which is then metabolised by KMO to produce kynurenine and subsequently 3-hydroxykynurenine. Ultimately, this leads to the production of quinolinic acid, an NMDA receptor agonist with neurotoxic properties. Alternatively, KATs produce kynurenic acid, a neuroprotective NMDA antagonist. This pathway is a key mechanism by which the balance between neuroprotection and neurotoxicity is maintained under stress [167,168].

Shaw et al. [169], Kaufman et al. [170] and Yunes et al. [171] have emphasised the pivotal role of gut bacteria in modulating host immune and neuroimmune responses by metabolising tryptophan via alternative enzymatic pathways, particularly the indole pathway. The bacterial enzyme tryptophanase, produced by species such as *Escherichia coli*, *Clostridium* and *Bacteroides*, converts tryptophan into indole. This can then be transformed into indole-3-propionic acid, indole-3-acetic acid and indole-3-aldehyde. These metabolites act as ligands for the aryl hydrocarbon receptor (AhR), a transcription factor that regulates mucosal immunity and neuroimmune communication. This links microbial metabolism directly to the host inflammatory response [169]. This signalling is further complemented by the direct microbial synthesis of neurotransmitters. For instance, certain *Lactobacillus* and *Bifidobacterium* strains utilise glutamate decarboxylase (GAD) to produce gamma-aminobutyric acid (GABA), which is a primary inhibitory neurotransmitter that modulates neuronal activity via GABA_A and GABA_B receptors. Other microbial enzymes are involved in synthesising dopamine, norepinephrine and acetylcholine, although their biochemistry remains poorly understood. Studies by Kaufman (1991) and Yunes (2016) have demonstrated that these microbial-derived metabolites can influence brain function in the host either via neuronal pathways, such as the vagus nerve, or through circulation [170,171]. This highlights the integrative role of the microbiota as a generator of signalling molecules and a regulator of host stress neurobiology, establishing it as a potential therapeutic target for stress-related disorders.

These mechanisms are particularly relevant in situations involving physical and physiological stress, such as illness, malnutrition, and prolonged exertion. This has been demonstrated by Dantzer et al. [172]. Stress-induced increases in intestinal permeability (‘leaky gut’) allow microbial products to enter the bloodstream and promote systemic inflammation, which can exacerbate fatigue, cognitive impairment and low mood. Saturio et al. [140], Tette et al. [163], Lei et al. [133], and Savulescu-Fiedler et al. [159] have emphasised the importance of understanding these molecular interactions to develop strategies that strengthen the intestinal barrier and promote a neuroprotective, anti-inflammatory microbial environment. Interventions such as dietary fibre, probiotics and prebiotics have been shown to increase the production of tight junction proteins (such as occludin and claudin), thereby reducing gut permeability and alleviating stress-induced systemic inflammation [9].

Research has demonstrated that certain bacterial genera and classes are consistently associated with positive or negative impacts on stress regulation, depending on their microbial taxonomy. For example, beneficial bacteria such as *Lactobacillus* and *Bifidobacterium* (classified in the *Bacilli* and *Actinobacteria* classes, respectively) are recognised for their ability to produce GABA and short-chain fatty acids, which have anti-inflammatory properties and can enhance mood [163,173]. Conversely, an overrepresentation of potentially harmful bacteria, including *Escherichia*, *Shigella* and certain *Clostridium* species (belonging to the classes *Gammaproteobacteria* and *Clostridia*), has been linked to increased intestinal inflammation, endotoxin production and altered tryptophan metabolism. Studies by Madison and Kiecolt-Glaser (2019) have shown that the balance between these microbial groups plays a critical role in determining the gut’s immune response and its ability to support or hinder central stress responses [174]. Notably, clinical investigations suggest that stress-induced dysbiosis is often accompanied by reductions in butyrate-producing bacteria, such as *Faecalibacterium prausnitzii*. This further weakens intestinal integrity and exacerbates HPA axis hyperactivity [175]. Consequently, identifying shifts in these microbial populations could be useful for both diagnosing and treating stress-related disorders.

Thus, these converging pathways illustrate the multifaceted role of the gut microbiome in shaping neuroimmune communication and stress adaptation, as analysed by Brun et al. [176]. The microbiome influences the function of the central nervous system by balancing serotonergic neurotransmission with neurotoxic or neuroprotective metabolites derived from the kynurenine pathway, indole-derived AhR ligands and direct microbial neurotransmitter production [167]. These metabolites can cross the blood–brain barrier and modulate synaptic plasticity, microglial activation and neurogenesis [177,178]. This demonstrates the broad reach of signals derived from the microbiome, establishing the gut microbiota as a central hub that integrates immune activation, enzymatic biochemistry and neurotransmitter signalling to influence the brain’s response to stress and susceptibility to neuropsychiatric disorders. Beneficial taxa such as *Lactobacillus* and *Bifidobacterium* can influence resilience versus vulnerability by modulating inflammatory signalling, neurotransmitter availability and gut barrier integrity, compared to pro-inflammatory strains such as *Escherichia* or *Clostridium*. These findings provide a rationale for microbiome-targeted interventions in stress-related conditions, supporting personalised, microbial profiling-guided approaches to optimise therapeutic outcomes [179].

### 5.2. The Microbiome–Vagus Nerve Axis and Its Neuroimmune and Metabolic Pathways

The PSNS and the gut microbiota work together to regulate stress resilience by modulating neural, hormonal and immune responses. One particularly well-defined mechanism involves pattern recognition receptors, notably Toll-like receptors (TLRs), which can identify conserved microbial molecules (PAMPs), as earlier studies have shown [180]. Studies have shown that the engagement of TLR4 by LPS, a component of the cell walls of Gram-negative bacteria, activates NF-κB signalling in intestinal dendritic cells and macrophages. This leads to the release of pro-inflammatory cytokines, including TNF-α, IL-1β and IL-6. These cytokines can enter the systemic circulation and reach the brain in two ways. Firstly, they can cross the blood–brain barrier (BBB) at leaky regions. Secondly, they can act on circumventricular organs. In both cases, elevated peripheral cytokine levels have been shown to increase CRH expression in the hypothalamus and potentiate HPA axis activation [181].

Another critical immunoregulatory pathway involves the aryl hydrocarbon receptor (AhR), which is a ligand-activated transcription factor that is expressed in various immune cell subsets, as a research team has demonstrated [182]. Microbial metabolism of tryptophan produces indole derivatives, including indole-3-aldehyde, which act as AhR ligands. AhR signalling activation in innate lymphoid cells (ILCs) has been shown to promote interleukin-22 (IL-22) secretion, a vital cytokine for maintaining epithelial barrier integrity. Increased IL-22 production strengthens mucosal defences, reducing microbial translocation and systemic immune activation. Studies in mice have demonstrated that those lacking the *AhR* gene exhibit increased gut permeability and heightened inflammatory responses, highlighting the protective role of this pathway in maintaining neuroimmune homeostasis [183].

Another key class of microbiota-derived metabolites with immunomodulatory properties is SCFAs, particularly butyrate and propionate. These SCFAs are recognised by G-protein-coupled receptors, such as GPR43 and GPR109A, which are found on T cells, dendritic cells and epithelial cells. Research has demonstrated that SCFAs promote the differentiation of regulatory T cells (Tregs), which are essential for maintaining immune tolerance. SCFAs mechanistically inhibit histone deacetylases (HDACs), leading to increased acetylation at the Foxp3 promoter region and the subsequent upregulation of this Treg-specific transcription factor [184]. The increased Treg population contributes to the suppression of systemic inflammation by secreting anti-inflammatory cytokines such as IL-10 and TGF-β [185]. These, in turn, moderate the stress axis, thereby reducing HPA hyperactivation.

As previously mentioned, the vagus nerve plays a key role in communication between the microbiome and the brain. Afferent fibres convey microbial and immune signals to the nucleus tractus solitarius (NTS), which regulates hypothalamic and limbic structures involved in stress responses [59]. Efferent vagal pathways mediate the cholinergic anti-inflammatory reflex by releasing acetylcholine, which binds to α7 nicotinic receptors on macrophages, thereby reducing TNF-α production [18]. This links gut microbial activity to central stress regulation and systemic immune tone.

In summary, the gut microbiota plays a pivotal role in immunoregulation, influencing cytokine profiles, barrier integrity, regulatory cell populations, and vagal signalling. This links peripheral inflammation with central neuroendocrine control. Disruption to this balance, caused by factors such as antibiotics, diet or stress, can trigger a feedback loop of inflammation and HPA axis dysfunction. This can contribute to the development of mood disorders, metabolic syndrome and autoimmune diseases [142].

### 5.3. Microbial Mediators of Neuroendocrine and Immune Crosstalk

Research has demonstrated that the integration of parasympathetic regulation with gut microbiota dynamics is widely accepted as a key factor in neuroimmune and metabolic homeostasis [37]. The peripheral nervous system, chiefly through vagal afferents, modulates intestinal function, immune signalling and microbial composition via the cholinergic anti-inflammatory reflex. The release of ACh from the vagal terminals activates α7 nAChRs on intestinal immune cells, particularly macrophages and dendritic cells. This leads to the suppression of pro-inflammatory cytokines through the inhibition of NF-κB [186]. This neural–immune signalling process serves to reduce local inflammation, thereby preserving the integrity of the epithelial barrier and supporting a symbiotic microbiota profile.

ChAT+ CD4+ T cells, which express choline acetyltransferase and are responsive to vagal input, act as key intermediaries by synthesising ACh within gut-associated lymphoid tissues, thereby propagating anti-inflammatory signals [187,188]. These T cells have been shown to influence AMP secretion, thereby shaping the microbial landscape by limiting the overgrowth of pathogenic bacteria and promoting microbial diversity. Furthermore, microbial metabolites such as SCFAs and polyamines reciprocally regulate host pathways, including Nrf2 activation, autophagy induction and mitochondrial metabolism. This fine-tunes the host’s immunometabolic tone. Dhawan et al. (2016) demonstrated that most ChAT-expressing T cells in the human and mouse intestine exhibit Th17-like characteristics [79]. These cells co-express the cytokines IL17A and IL22, as well as the transcription factor RORC. Their generation is also influenced by dendritic cells following activation of the β2-adrenergic receptor. Studies in CD4ChAT^−/−^ mice, which lack ChAT specifically in CD4+ T cells, showed reduced levels of epithelial antimicrobial peptides (AMPs), such as lysozyme, defensin A and angiogenin 4. This was accompanied by increased bacterial diversity. This indicates that ChAT-expressing T cells in the gut are stimulated via adrenergic receptor signalling in dendritic cells. These cells play a key role in regulating antimicrobial peptide secretion and microbial homeostasis.

As discussed previously, parasympathetic input is crucial for regulating intestinal motility, secretion, and permeability [189], which in turn shapes the microbial habitat and nutrient availability. Dysregulation, as observed in conditions such as chronic stress or autonomic neuropathy, disrupts gut–brain communication, thereby promoting dysbiosis, chronic inflammation and metabolic dysfunction [3]. Vagal afferents transmit microbial and immune signals to the nucleus tractus solitarius, influencing the hypothalamic and limbic circuits that govern stress and emotional behaviour [18]. This highlights the potential of parasympathetic signalling as a target for bioelectronic and microbiota-directed therapies.

A complex network of interconnected molecular pathways serves as the regulatory system for neuroimmune function, metabolic adaptation and host–microbiota interactions. NO is produced via both classical nitric oxide synthase (NOS)-dependent pathways and microbiome-mediated nitrate–nitrite reduction. It exerts pleiotropic effects on vascular tone, mitochondrial respiration, and immune modulation [190]. It has been demonstrated that NO significantly inhibits pro-inflammatory signalling, particularly by suppressing NF-κB activation. Furthermore, NO has been observed to synergise with antioxidant responses that are co-ordinately regulated by Nrf2, a redox-sensitive transcription factor that governs the expression of cytoprotective genes such as HO-1 and NQO1. Activation of Nrf2 also intersects with autophagy and lysosomal pathways, which are essential for cellular quality control, antigen processing and the removal of damaged mitochondria via mitophagy [191]. These degradative systems maintain immune tolerance and metabolic efficiency, particularly under inflammatory or oxidative stress.

At the same time, the cholinergic anti-inflammatory reflex works by stimulating the vagus nerve. This releases ACh from efferent fibres or ChAT+ T cells. The ACh then binds to α7 nAChRs on immune cells. This reduces cytokine release and prevents the overactivation of NF-κB [41]. Other neurotransmitter systems, including the GABAergic and serotonergic axes, also modulate immune responses. GABA limits inflammatory signalling and promotes regulatory phenotypes, while serotonin regulates immune cell trafficking and mucosal defence, partly via interactions with the gut microbiota. Additionally, microbial metabolites such as tryptophan catabolites act as precursors for peripheral serotonin synthesis, thereby strengthening the role of the microbiome in linking metabolic activity with neuroimmune signalling [192,193].

Furthermore, polyamine metabolism, particularly that of spermidine, has been shown to promote cellular resilience by inducing autophagy and modulating T cell function, as demonstrated in a study by Ning et al. [194]. Additionally, it has been established that peroxisome proliferator-activated receptor gamma (PPARγ), a nuclear receptor, functions as a pivotal transcriptional regulator of lipid metabolism and inflammation. Upon activation, it represses NF-κB-mediated transcription, enhances mitochondrial biogenesis and promotes anti-inflammatory (M2) macrophage polarization, thus coupling metabolic rewiring with immune homeostasis [195]. Together, these pathways form a dynamic network that enables the organism to respond to internal and external stressors by coordinating immune responses, metabolic flexibility and microbial symbiosis.

In summary, immune–metabolic homeostasis is governed by nitric oxide signalling, Nrf2-mediated redox regulation, lysosomal–autophagic processes, and cholinergic neuroimmune pathways. They regulate inflammation, oxidative stress and mitochondrial quality. The cholinergic anti-inflammatory reflex and GABAergic/serotonergic modulation support neuroimmune communication at barrier sites, such as the gut [196]. Metabolic regulators, including polyamines and PPARγ, also help to maintain immunometabolic balance. This provides a molecular framework that integrates nervous, immune, and microbial signals in order to sustain physiological resilience.

### 5.4. Parasympathetic Modulation of GABAergic and Serotonergic Signalling in the Gut–Brain Axis

As demonstrated earlier, the PSNS, primarily via the vagus nerve, mediates bidirectional communication between the gut microbiota and the CNS, integrating GABA and serotonin signalling to shape neurochemical, immune and stress responses. The presence of GABA-producing microbes, such as *Lactobacillus* and *Bifidobacterium* species, in the gastrointestinal tract can influence CNS function via the vagus nerve, which contains GABA receptors that respond to neurotransmitters and peptides found in the lumen [171]. GABA_A and GABA_B receptors on vagal sensory neurons modulate their firing rates, thereby relaying microbial and metabolic signals to the nucleus tractus solitarius and other central autonomic nuclei. This afferent input then influences the hypothalamic regulation of the HPA axis and modulates the activity of GABAergic interneurons in stress-sensitive regions, such as the amygdala and prefrontal cortex. It should also be noted that impaired vagal afferent transmission can weaken GABAergic inhibition, thereby enhancing excitatory drive in these brain regions [197].

Furthermore, activating the vagus nerve enhances central GABAergic tone, reducing excitotoxicity and facilitating the inhibitory control necessary for adaptive plasticity. Animal studies have demonstrated that vagal integrity is essential for the anxiolytic and antidepressant effects of GABA-producing probiotics, highlighting the parasympathetic dependency of this gut-to-brain modulatory loop [163]. Clinical studies also suggest that vagus nerve stimulation may replicate some of these effects, supporting its use as an adjunctive treatment for refractory depression and anxiety disorders [198,199].

The PSNS exerts a significant influence on serotonergic signalling at multiple levels. Firstly, vagal activity modulates the secretion of 5-HT from enterochromaffin cells in the gut mucosa. These cells are responsible for producing over 90% of the body’s total serotonin supply [200]. This process is partly driven by microbial-derived SCFAs and bile acids, the action of which on EC cells is transmitted through vagal pathways. Secondly, vagal input affects central serotonergic circuits, particularly the raphe nuclei. These nuclei regulate emotional behaviour and synaptic plasticity via 5-HT1A and 5-HT3 receptors. The activation of 5-HT1A receptors, which is facilitated by vagal tone and microbial signalling, has been shown to promote neurogenesis and dendritic remodelling via BDNF-dependent pathways. These pathways are crucial for maintaining resilience under chronic stress [201]. Conversely, 5-HT_3_ receptors, which are located on vagal afferents and brainstem circuits, modulate rapid synaptic responses to visceral and affective stimuli. This links gut-derived serotonin to the rapid perception of stress and pain [59,202]. Thus, the vagus nerve operates as a functional bridge between peripheral and central serotonergic pools, as well as between inhibitory and excitatory neuromodulatory systems.

The GABAergic and serotonergic systems provide the neurochemical basis through which the parasympathetic nervous system affects the whole body [203]. Vagal modulation enhances inhibitory neurotransmission, reduces activation of the HPA axis and promotes brain plasticity in response to stress. However, vagal dysfunction, induced by factors such as chronic inflammation, metabolic imbalance or psychological stress, disrupts these pathways. This increases vulnerability to mood disorders and impairs gut–brain homeostasis [10]. Therefore, maintaining the functional integrity of the PSNS is crucial for effective microbiota-driven GABAergic and serotonergic signalling. This signalling influences stress adaptation, emotion regulation and neuroplasticity via vagal afferent pathways [10,131]. Consequently, therapeutic strategies targeting vagal tone, such as probiotics, dietary interventions, biofeedback and direct vagus nerve stimulation, show promise in restoring gut–brain homeostasis and treating conditions associated with gut–brain dysregulation, including depression, anxiety and functional gastrointestinal disorders [18,204].

## 6. Pathophysiology and Disease Implications

### 6.1. Consequences of Parasympathetic Dysfunction and Microbial Imbalance

Among neuropsychiatric disorders, major depressive disorder (MDD), generalised anxiety disorder (GAD), autism spectrum disorder (ASD) and Alzheimer’s disease (AD) have been found to be associated with vagal hypoactivity, altered gut microbial diversity, neuroinflammation and disturbed tryptophan metabolism [130,205,206]. In the context of functional gastrointestinal disorders (FGIDs), including irritable bowel syndrome (IBS), functional dyspepsia and small intestinal bacterial overgrowth (SIBO), mounting evidence links vagal withdrawal and gut dysbiosis to increased visceral sensitivity, impaired motility and low-grade intestinal inflammation (Table 2). Such findings suggest that central nervous system pathology and peripheral gastrointestinal dysfunction may converge on a common mechanism of vagal impairment.

The PSNS, particularly via the vagus nerve, plays a central role in regulating the gut microbiota and maintaining homeostasis between the host and microbiome. This is achieved through bidirectional communication along the microbiota–gut–brain axis, involving neural, endocrine, and immune pathways [130,205]. Parasympathetic activity influences gastrointestinal motility, secretion and local immunity, thereby shaping the microbial environment. In contrast, reduced vagal tone has been linked to increased intestinal permeability, systemic endotoxin load and pro-inflammatory cytokine activation. This exacerbates neuroinflammatory processes and perpetuates systemic inflammation and dysbiosis, which can contribute to the development of psychiatric and gastrointestinal disorders. Interventions that restore vagal activity, such as lifestyle changes, dietary modifications, probiotics or vagus nerve stimulation, may therefore offer therapeutic benefits [11,18].

### 6.2. Vagal–Microbiota Interactions in Neuropsychiatric, Metabolic and Inflammatory Disorders

Clinical studies have provided evidence that modulation of the gut microbiota through probiotics, diet and microbiota-targeted therapies can affect neuropsychiatric outcomes via serotonergic and immune mechanisms, which may be mediated by parasympathetic pathways. For example, *Bifidobacterium breve* CCFM1025 has been shown to alleviate depressive symptoms by restoring tryptophan metabolism and microbial diversity. This suggests a functional association between the microbiota and the serotonergic system, which is likely to involve vagal afferents [207]. Similarly, multi-strain probiotics have been observed to reduce gastrointestinal and emotional symptoms in patients diagnosed with major depressive disorder, further supporting the concept of a gut–brain regulatory loop [206,208]. Moreover, altered vagal activity has been identified as a predictive biomarker of treatment response in depressive and anxiety disorders, indicating that parasympathetic tone could serve as both a mediator and a clinical marker [219,220].

Cardiometabolic diseases, including hypertension, metabolic syndrome and type 2 diabetes mellitus, are associated with reduced parasympathetic tone and systemic inflammation. These diseases are often accompanied by microbial translocation and endotoxemia, which further impair metabolic regulation [142]. Immune and inflammatory diseases, including rheumatoid arthritis, inflammatory bowel disease and multiple sclerosis, have been linked to disrupted vagal anti-inflammatory signalling and loss of microbial immune tolerance. Accumulating evidence suggests that impaired vagal signalling may facilitate a pro-inflammatory environment by enhancing Th17 responses and reducing regulatory T-cell activity [18,221]. There is now an emerging interest in vagus nerve stimulation and microbiota-targeted therapies, such as probiotics, prebiotics, and potential modulators of the disease’s progression. Finally, post-infectious syndromes such as myalgic encephalomyelitis, chronic fatigue syndrome and post-COVID-19 dysautonomia exhibit co-occurring vagal dysfunction and microbiome alterations. These result in autonomic instability, mitochondrial stress and persistent low-grade inflammation [222]. These overlapping mechanisms highlight the unifying role of vagal–microbial interactions across psychiatric, metabolic, inflammatory and post-infectious conditions.

Experimental models have confirmed the involvement of the parasympathetic system in microbiome-mediated immunomodulation. For example, in a murine model of multiple sclerosis, intermittent fasting resulted in favourable changes to microbial communities and reduced pro-inflammatory T-cell responses. These effects could be transferred through faecal microbiota transplantation, indicating that changes in the microbiota can modulate neuroimmune functions via parasympathetic mechanisms [217]. Similarly, in metabolic syndrome, analogous interventions have been shown to improve both cardiometabolic outcomes and microbial composition, indicating a systemic parasympathetic influence on metabolic and microbial regulation [218]. Further studies have also demonstrated that restoration of vagal signalling enhances mitochondrial bioenergetics and reduces oxidative stress, both of which are critical factors for resilience in chronic stress-related disorders [223].

These findings therefore underscore the importance of parasympathetic regulation in shaping the gut microbiome and mediating its effects on host physiology. As research progresses, therapies targeting parasympathetic pathways—including vagus nerve stimulation, behavioural strategies and personalised nutrition—may offer a promising way to restore the balance between the microbiota and the host in cases of neuropsychiatric, metabolic and inflammatory conditions [205,212,216]. Importantly, such interventions address not only the symptoms but also the underlying pathophysiological mechanisms, offering the potential for disease-modifying effects. These results highlight the importance of considering both parasympathetic and microbial factors in the pathogenesis, diagnosis and treatment of chronic disorders.

### 6.3. Cellular Mechanisms: Lysosomal Function, Autophagy and Microbial Homeostasis

A substantial body of research has demonstrated that lysosomes are multifunctional organelles that degrade macromolecules and act as signalling hubs that integrate environmental cues, including those derived from the gut microbiota [224]. As well as their well-established role in breaking down bacterial products and cellular debris, it has been shown that lysosomes can regulate immune responses via pathways such as mTORC1, TFEB-mediated transcriptional programmes and lysosomal calcium signalling. Microbial metabolites, particularly SCFAs such as butyrate, have been shown to enhance lysosomal biogenesis and acidification by modulating histone acetylation and TFEB nuclear translocation [225]. Furthermore, dysbiosis-induced alterations in bile acid profiles have been shown to disrupt lysosomal lipid metabolism, resulting in the accumulation of cholesterol and lipofuscin. This, in turn, impairs lysosomal enzymatic function. This dysfunction is strongly associated with chronic inflammation and impaired antigen presentation via MHC class II molecules [226]. In intestinal macrophages, defective lysosomal activity compromises bacterial clearance and promotes a pro-inflammatory phenotype, thereby sustaining mucosal inflammation. Importantly, lysosomal signalling also interfaces with mitochondrial quality control via mitophagy, linking organelle integrity to systemic immune responses [227,228]. Notably, certain gut bacteria, such as *Bacteroides fragilis*, have been observed to directly modulate lysosomal function by secreting polysaccharide A, which enhances regulatory T-cell activity and immunological tolerance [229].

In particular, autophagy and its selective forms, such as xenophagy and mitophagy, play a pivotal role in maintaining mucosal barrier integrity and regulating intracellular bacterial populations [230]. This cellular process is closely associated with lysosomal degradation and is influenced by factors including nutrient status and microbial signals. Gut-derived factors, including SCFAs and polyamines such as spermidine, activate autophagy by stimulating AMPK and inhibiting mTORC1 [231]. However, bacterial pathogens, including *Salmonella* and *Shigella*, have evolved mechanisms to evade or subvert autophagy targeting [232]. Genetic variants in autophagy-related genes, notably the ATG16L1 T300A polymorphism, have been shown to impair Paneth cell granule exocytosis and antimicrobial peptide release. This alters microbiota composition and promotes dysbiosis, as demonstrated by Cheng et al. [233]. Furthermore, impaired autophagy has been shown to disrupt antigen processing and cross-presentation, thereby compromising mucosal immunity. Recent findings indicate that impaired autophagy in dendritic cells and macrophages results in uncontrolled inflammasome activation and IL-1β secretion, further driving chronic gut inflammation [234]. This suggests that autophagy is not only a degradative pathway but also a crucial regulator of immune tolerance and microbiota symbiosis. Notably, therapeutic strategies aimed at restoring autophagy flux are being investigated as potential interventions for inflammatory bowel disease and metabolic disorders linked to microbiota imbalance [235]. These strategies include the use of pharmacological agents such as rapamycin and resveratrol, as well as microbial-derived compounds [236].

Building on these findings, it is equally important to consider the role of neuroimmune pathways in maintaining gut–brain–immune homeostasis. Whether the cholinergic anti-inflammatory reflex in the gut–brain–immune axis exists in a fully conserved manner across species remains to be elucidated. This pathway constitutes a neuroimmune feedback loop that modulates systemic and local inflammation via the parasympathetic nervous system, primarily through the vagus nerve [10]. The gut microbiota exerts a profound influence on this reflex by modulating vagal afferent sensitivity, neurotransmitter biosynthesis and central autonomic output. Certain commensal species, such as *Lactobacillus reuteri* and *Bifidobacterium longum*, have been shown to enhance vagal tone and increase the expression of ACh-synthesising enzymes in enteric neurons [132,237].

The ACh released via vagal afferents binds to α7nAChRs on macrophages, dendritic cells and microglia. This suppresses NF-κB-mediated transcription of pro-inflammatory cytokines, such as TNF-α and IL-6 [45]. This receptor-mediated signalling also inhibits NLRP3 inflammasome assembly, thereby reducing caspase-1 activation and IL-1β maturation, as demonstrated by Chen et al. [41]. Han et al. (2019) demonstrated that the presence of *Lactobacillus mucosae* NK41 and *Bifidobacterium longum* NK46 significantly alleviates symptoms of anxiety, depression and colitis induced by immobilisation stress [238]. This alleviation is achieved through modulation of gut dysbiosis and inflammatory markers. These agents reduce the activation of NF-κB in the hippocampus, enhance the expression of brain-derived neurotrophic factor (BDNF) and decrease the levels of pro-inflammatory cytokines and gut bacterial lipopolysaccharides. These data provide a mechanistic explanation for the psychobiotic effects of probiotics, linking vagal cholinergic signalling to improved neuroplasticity and immune tolerance.

Concurrently, the microbiota modulates pivotal tryptophan-kynurenine metabolic pathways, thereby influencing CNS inflammation and neuroplasticity [239]. Metabolites such as indole derivatives and GABA, produced by gut microbes, may act synergistically with vagal activation to regulate neuroimmune communication [240]. Taken together, these findings emphasise the pivotal role of the gut microbiota in modulating the gut–brain–immune axis, with potential implications for treating sepsis, IBD, and neuroinflammatory diseases such as multiple sclerosis and Parkinson’s disease [130].

**Figure 6:**
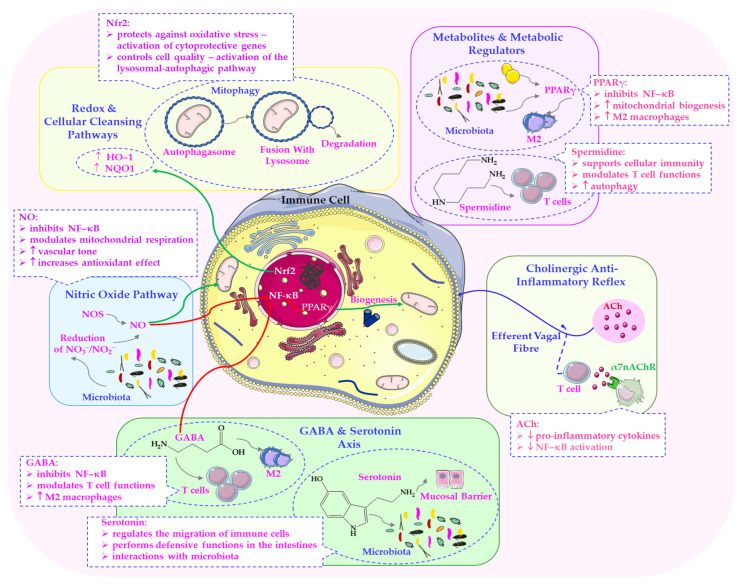
**Integrated neuroimmune–metabolic network in host–microbiota homeostasis.** This network involves the modulation of inflammation (via NF-κB inhibition), the maintenance of redox balance (via Nrf2, or nuclear factor erythroid 2-related factor 2) and the control of mitochondrial quality (autophagy/mitophagy) through nitric oxide (NO) signalling, via both NOS-dependent (nitric oxide synthase) and microbiota-mediated pathways. The cholinergic anti-inflammatory reflex (α7 nAChR; alpha-7 nicotinic acetylcholine receptor), GABAergic and serotonergic neurotransmission, polyamine metabolism (spermidine) and the nuclear receptor PPARγ (peroxisome proliferator-activated receptor gamma) coordinate immune tolerance, metabolic adaptation and microbiota–host interactions, particularly under oxidative or inflammatory stress. **Abbreviations:** ACh—acetylcholine; α7nAChRs—α7 nicotinic acetylcholine receptors; GABA—γ-aminobutyric acid; HO-1—heme oxygenase-1; NF-κB—nuclear factor-kappa B; NO—nitric oxide; Nrf2—nuclear factor erythroid 2-related factor 2; NQO1—NAD(P)H:quinone oxidoreductase 1; NOS—nitric oxide synthase; PPARγ—peroxisome proliferator-activated receptor gamma. This figure was created using Servier Medical Art (available at https://smart.servier.com/) (accessed on 1 May 2025).

Figure 6 shows the integrated neuroimmune–metabolic network that maintains homeostasis between the host and microbiota. It shows how neural, immune and metabolic pathways interact with microbial communities to regulate physiological balance, energy metabolism and adaptive responses to environmental and internal stressors.

In summary, it is clear that the gut microbiota influences immune responses and maintains host homeostasis through the pivotal molecular mechanisms of lysosomal function, autophagy, and the cholinergic anti-inflammatory reflex. The impact of microbial metabolites and structural components on lysosomal signalling, autophagy and vagus nerve-mediated anti-inflammatory pathways is crucial in modulating both local and systemic inflammation. When these mechanisms become dysregulated, the resulting imbalance contributes to local intestinal pathology and systemic conditions including metabolic syndrome, autoimmune diseases and neurodegeneration [18,241]. Dysregulation of these processes, which is often associated with dysbiosis, plays a role in the development of inflammatory, metabolic and neurodegenerative diseases.

## 7. Clinical Implications and Therapeutic Opportunities

### 7.1. Emerging Therapies: Neuromodulation, Probiotics, Prebiotics, and Lifestyle Optimisation

Due to its central role in the nervous, immune and microbial systems, the vagus nerve is a promising therapeutic target. Non-invasive vagus nerve stimulation, such as transcutaneous auricular stimulation, has been shown to reduce inflammatory biomarkers, improve mood and restore autonomic balance in conditions including epilepsy, depression, migraine and irritable bowel syndrome [242], likely via enhanced cholinergic anti-inflammatory signalling, HPA axis modulation and improved gut microbiota composition [243,244]. Indirect interventions, such as mindfulness-based stress reduction, diaphragmatic breathing and prebiotic/probiotic supplementation, provide accessible ways to boost vagal tone and resilience to chronic stress [245,246,247]. There is emerging evidence that combining vagal stimulation with targeted microbiome modulation could provide synergistic benefits by influencing neuroimmune pathways, microbial metabolite production (e.g., SCFAs and GABA) and central neurotransmitter signalling simultaneously [89]. Future research should personalise vagal modulation therapies according to individual vagal phenotypes, microbiota profiles, and disease-specific biomarkers. This will advance integrative approaches in neurogastroenterology and psychoneuroimmunology, with the potential to alleviate symptoms, restore systemic homeostasis, and prevent the progression of chronic conditions.

Therapeutic strategies that modulate the parasympathetic nervous system and the gut microbiome are emerging as promising tools for mitigating the physiological and psychological effects of stress. These strategies target individual mechanisms while combining multiple complementary approaches. Neuromodulatory interventions, including both invasive and non-invasive vagus nerve stimulation, have been demonstrated to increase parasympathetic activity, decrease systemic inflammation and improve autonomic balance [248]. This effectively links neural regulation with immune and metabolic homeostasis in a quantifiable manner [249]. These interventions may also indirectly influence the composition of the gut microbiome through modulation of vagal afferent and efferent signalling, thereby impacting microbiota-mediated neuroimmune pathways. Alongside these approaches, microbiome-targeted strategies involving the use of carefully selected probiotics and prebiotics support gut–brain communication, modulate neuroimmune pathways and maintain microbial balance. This contributes to enhanced stress adaptation and resilience [250,251]. Optimising lifestyle factors, including structured exercise programmes, tailored dietary modifications, sleep hygiene and IHT, can further reinforce these effects by improving vagal activity, metabolic flexibility and microbiome composition and function [252,253]. Therefore, integrating neuromodulation, microbiome-targeted therapies, and lifestyle interventions establishes a synergistic, multidimensional strategy to enhance stress resilience via convergent neural, immune, and metabolic pathways [254,255].

Meanwhile, mounting evidence indicates that IHT may impact the gut microbiota via NO-mediated signalling pathways, redox homeostasis, and modulation of inflammatory responses. A study by Van Meijel et al. (2022) showed that mild intermittent hypoxia exposure in overweight and obese men induces modest shifts in the composition of the gut microbiota, resulting in an increased abundance of anaerobic, butyrate-producing bacteria [121,122]. These microbial changes were associated with alterations in glucose and lipid metabolism, suggesting a potential link between mild intermittent hypoxia, modulation of the gut microbiota, and the regulation of the host’s metabolism.

It is important to distinguish between mild, controlled IHT, which has potential therapeutic effects, and chronic, severe intermittent hypoxia, as observed in obstructive sleep apnoea (OSA), which has well-documented pathological consequences. However, studies of intermittent hypoxia in OSA patients have demonstrated that hypoxic stress can cause dysbiosis of the gut microbiota. This is evidenced by reduced microbial diversity and decreased SCFA production [124]. These findings suggest that the severity of oxygen desaturation correlates with microbiota dysbiosis, emphasising the importance of monitoring hypoxia-related parameters in OSA management. The study by Moreno-Indias et al. (2015) also showed that chronic intermittent hypoxia in a mouse model of sleep apnoea significantly alters the composition of the gut microbiota, increasing overall diversity and shifting the balance at the phylum level towards a higher proportion of *Firmicutes* and a lower proportion of *Bacteroidetes* and *Proteobacteria* [256]. These results suggest that the hypoxia-reoxygenation patterns typical of OSA may disrupt interactions between the host and microbiota and lead to metabolic changes associated with the disease. This emphasises the dual nature of intermittent hypoxia: while controlled IHT can be beneficial, chronic pathological hypoxia contributes to dysbiosis and systemic inflammation.

Furthermore, lifestyle factors such as physical activity and emotional well-being have been shown to shape the gut microbiome independently, suggesting a convergent mechanism whereby IHT interacts with behavioural and psychological factors to enhance microbial and systemic homeostasis [120]. Collectively, this evidence supports a model in which, when applied in a controlled, health-promoting context, intermittent hypoxia modulates key regulatory networks, including mitochondrial function, autonomic balance and gut microbiota composition, thereby enhancing resilience to stress and promoting integrated physiological health.

### 7.2. Personalised Approaches: Integrating Physiology, the Microbiome and Patient-Specific Stress Profiles

Personalised approaches to stress modulation recognise that people differ greatly in terms of their physiology, the composition of their gut microbiome, and their sensitivity to stress. These factors critically influence the effectiveness of any intervention [257]. Emerging evidence suggests that variability between individuals in terms of vagal tone, microbial diversity and metabolic responses can have a significant impact on the outcomes of neuromodulatory, dietary and lifestyle interventions [258].

In our works, we have examined differences in hypoxia tolerance across animal models and human studies to demonstrate these personalised strategies, showing how variability in physiological and biochemical responses can inform targeted interventions [103,104,259,260,261]. Integrating autonomic nervous system assessments, such as parasympathetic tone and vagal activity, alongside microbiome profiling and stress response characteristics, allows interventions to be designed that are tailored to each subject’s specific needs and engage the most relevant mechanisms. This approach also enables the identification of potential biomarkers of responsiveness, such as SCFA levels, inflammatory cytokine profiles and stress hormone dynamics. These biomarkers can then be used to guide personalised treatment [98,262,263,264].

Both neuronal and non-neuronal ACh systems act in a tightly coordinated manner to regulate immune, metabolic and neurophysiological responses. The vagus nerve mediates parasympathetic signalling and interacts with the immune and gut systems. Meanwhile, non-neuronal ACh, which is produced by epithelial and immune cells, contributes to the local modulation of inflammation and oxidative stress. Advanced glycation end products (AGEs), which are formed through the non-enzymatic reaction of reducing sugars with amino groups, can trigger oxidative stress and inflammation by interacting with RAGE (receptors for AGEs). This links carbonyl stress to neurodegenerative processes. Dietary polyphenols exert protective effects by scavenging reactive oxygen species, inhibiting AGE formation, blocking RAGE–ligand interactions and modulating the gut microbiota. This supports the integrated function of the neuronal and non-neuronal ACh systems, offering potential neuroprotection [265]. This framework enables the development of multidimensional strategies to enhance vagal modulation, support microbial homeostasis and strengthen resilience to stress. Personalised interventions may include customised dietary plans, targeted prebiotic or probiotic supplementation, lifestyle adaptations and neuromodulatory techniques, such as vagus nerve stimulation or controlled intermittent hypoxia training, where appropriate [263,266]. Combining these interventions according to individual physiological and microbial profiles can optimise neuroimmune and metabolic outcomes, improve stress adaptation, and reduce susceptibility to neuropsychiatric and metabolic disorders [49,99]. This approach is both predictive and preventive and adapted to each individual’s unique neuroimmune, metabolic and hypoxia response profile. It provides a mechanistically grounded, patient-centred strategy for enhancing resilience and long-term health.

Several controversies and open questions remain in the field of personalised gut–brain axis research, which integrates physiology, the microbiome and patient-specific stress profiles. The effects of specific probiotic strains can vary significantly depending on factors such as strain, dose, experimental model, and population. This highlights the need for careful interpretation and comparison of results from different studies [265,267]. Similarly, the therapeutic efficacy of non-invasive vagus nerve stimulation remains debated, with inconsistent outcomes attributed to differences in methodology, stimulation parameters and populations. Other unresolved questions include how sex, age, lifestyle, and other individual factors modulate the gut–brain axis and potentially shape interindividual differences in stress resilience. Our review critically analyses and synthesises these findings to emphasise the importance of integrative, patient-specific strategies that consider physiological and microbial factors alike. This identifies areas for future research to optimise personalised interventions.

## 8. Conclusions and Future Perspectives

The findings presented here make a significant contribution to our scientific understanding of the complex regulatory system that governs how cells adapt to physiological and environmental stressors. Of particular interest is the central role of ACh within this network. By acting on calcium signalling, NO pathways and mitochondrial bioenergetics, ACh acts as both a neurotransmitter and a metabolic regulator. Its influence extends beyond synaptic transmission to encompass mitochondrial respiratory control, redox homeostasis and modulation of systemic inflammatory responses. Recent evidence also suggests that ACh may regulate autophagy and lysosomal function, thereby linking neurotransmission further with cellular stress adaptation.

It has been demonstrated that the PSNS, and more specifically the vagus nerve, plays a pivotal role in enhancing stress resilience. This is achieved by modulating the HPA axis, inflammation, and gut–brain communication. IHT has been shown to enhance vagal tone and increase NO availability, thereby supporting autonomic balance and anti-inflammatory effects. Notably, under hypoxic conditions, microbiota-mediated nitrate reduction emerges as a significant alternative source of NO, establishing a link between gut microbial activity and systemic adaptation. Furthermore, IHT has been shown to promote mitochondrial flexibility by activating HIF-1α and Nrf2, thereby enhancing resistance to oxidative stress and improving energy efficiency. IHT has also been observed to stimulate mitophagy and improve NO-mediated vascular responses, ensuring tissue perfusion during oxygen fluctuations. This multifaceted response highlights IHT’s capacity to modulate autonomic and metabolic systems, as well as reshape gut microbial ecology, thereby strengthening host–microbiome resilience under stress.

The reciprocal relationship between the composition of the gut microbiota and the host’s physiological system introduces an additional layer of intricacy. The microbiome influences central neurotransmitter systems, including the GABAergic and serotonergic pathways, by contributing to the bioavailability of metabolic intermediates such as nitrates and polyamines. These interactions have been shown to affect emotional regulation, neuroplasticity and behavioural responses to stress. Moreover, alterations in microbial metabolites, such as SCFAs and indole derivatives, have been directly linked to vagal signalling and central neuroimmune modulation. This reinforces the concept of the gut–brain–immune axis as a unified regulatory network. As demonstrated, individual physiological reactivity, including susceptibility to bioenergetic hypoxia, is closely linked to mitochondrial performance and microbiome composition.

Although targeting the parasympathetic nervous system and gut microbiota shows promise, inter-individual variability must be considered. Future research should use longitudinal, multi-omics approaches that combine microbiome sequencing, metabolomics, and vagal tone assessment. This will allow us to progress from correlation to causation and enable us to develop mechanistically informed, patient-specific interventions.

Thus, the convergence of cholinergic, metabolic and microbiome-mediated pathways provides a comprehensive view of stress regulation. Adopting this integrative approach could advance our understanding of stress physiology and inform more personalised and effective therapeutic strategies. This has significant clinical implications, and further research in this area is required. Modulating the PSNS and gut microbiota through vagal nerve stimulation, dietary interventions, or probiotics represents a promising therapeutic approach for managing stress-related disorders. These approaches could enhance stress resilience, restore neuroimmune balance and improve metabolic efficiency in patients with anxiety, depression and related chronic diseases. Future research should prioritise identifying biomarkers of individual adaptive capacity (e.g., vagal tone indices, microbiota-derived metabolites, and mitochondrial stress markers) and developing personalised therapeutic algorithms tailored to patient-specific profiles.

## 9. Limitations

Despite the integrative framework presented, it is important to acknowledge that the current findings are limited by the constraints of the experimental design and conceptual extrapolation. Much of the evidence supporting the roles of acetylcholine, nitric oxide signalling and mitochondrial adaptation comes from preclinical studies or small-scale human trials. This restricts the generalisability of these results to populations with diverse genetic, environmental and lifestyle characteristics.

Furthermore, the bidirectional interactions between the vagus nerve, mitochondrial bioenergetics, and the gut microbiota have not been fully characterised at a mechanistic level, as existing data are often correlative rather than causative. The heterogeneity of microbiome composition between individuals, coupled with the dynamic nature of microbial metabolism, introduces additional variability that complicates the translation of controlled experimental observations into real-world clinical contexts.

Despite providing comprehensive insights, the current evidence remains heavily preclinical, so it is not yet possible to establish firm correlations or causation. In addition, the significant variability in microbiome composition and vagal tone observed among individuals poses a significant challenge to the translation of findings into personalised interventions [268]. This variability makes it difficult to predict therapeutic responses and highlights the importance of individualised assessment when applying neuroimmune–microbiome-targeted strategies.

Additionally, reliance on biomarkers of vagal tone or nitric oxide bioavailability may obscure the nuanced temporal and spatial dynamics of these pathways. Another important limitation is the scarcity of large-scale randomised controlled clinical trials that could validate the efficacy and safety of interventions such as intermittent hypoxia training, vagus nerve stimulation or microbiome-targeted therapies in stress-related conditions, highlighting the need for longitudinal, multi-omic and systems-level approaches that can capture the complexity of autonomic regulation and stress resilience in vivo.

## Figures and Tables

**Table 1:** A summary of selected studies examining the role of gut microbiota and parasympathetic signalling in the gut–brain and gut–organ axes.

	Study Models	Methods and Doses	Key Findings	Molecular Mechanisms	References
1	Transgenic mice (Glp1r[GCamp6]) with abdominal window for in vivo imaging of Brunner’s glands	Cholecystokinin injection at 10 µg/kg; vagotomy and sensory denervation used to assess vagal role	Stress suppresses vagal activity → reduces Brunner’s gland secretion → alters microbiome (↓ *Lactobacillus*)	Stress inhibits central amygdala → lowers dorsal motor nucleus of the vagus activity → reduces parasympathetic output to duodenum → modifies the gut microbiota composition	[141]
2	Rats on high-fat diet (HFD) for 3 days or 4 weeks; induced metabolic disturbances resembling insulin resistance and hyperphagia model	Acetate infusion (2, 8, or 20 µmol/kg/min); metabolic assessments	Microbiota-derived acetate activates vagus nerve → ↑ insulin secretion, ↑ ghrelin, ↑ appetite → metabolic syndrome	Acetate stimulates parasympathetic signalling to β-cells → promotes hyperinsulinemia and obesity	[142]
3	Review of animal and human studies on the gut–microbiota–brain axis	Systematic literature review; no experimental interventions or dosing	Gut microbiota influences brain function through multiple systems including the autonomic nervous system, especially parasympathetic pathways	Parasympathetic signalling via the vagus nerve is one of five key routes linking the gut microbiota to the brain, along with neuroendocrine, immune, neurotransmitter, and barrier mechanisms	[143]
4	Human case–control study comparing fecal microbiota in 72 Parkinson’s disease patients and 72 healthy controls	16S rRNA gene pyrosequencing (V1–V3 regions); statistical analysis using generalized linear models. No treatment or dosing applied	Parkinson’s disease patients had a 77.6% reduction in *Prevotellaceae* abundance. Decreased *Prevotellaceae* was associated with PD diagnosis, while *Enterobacteriaceae* abundance correlated with postural instability and gait difficulty	Altered gut microbiota may influence disease through interactions with the enteric nervous system and vagus nerve, both early targets of α-synuclein pathology in Parkinson’s disease	[2]
5	Mice with hepatocellular carcinoma; liver vagotomy, CD8+ T cell manipulation, microbiota transfer	Hepatic vagotomy; pharmacologic vagal activation; CD8+ T cell depletion; Chrm3 knockout; microbiota transplantation from HCC donors	Vagotomy → ↓ liver tumor growth, ↓ fatigue, ↓ anxiety. Vagal stimulation → ↑ tumor progression via immune suppression. Microbiota from HCC donors → impaired behavior and immunity	Vagal acetylcholine → CHRM3 receptor on CD8+ T cells → ↓ anti-tumor immunity. Gut microbiota + vagus nerve → regulate liver immune response and behavior	[6]
6	Mice lacking the α7 nicotinic acetylcholine receptor gene (Chrna7 knock-out), compared to wild-type mice. Subdiaphragmatic vagotomy was performed to investigate vagus nerve involvement	Subdiaphragmatic vagotomy; behavioral tests for depression; linear discriminant analysis effect size microbiota analysis; plasma metabolomics; synaptic protein analysis in medial prefrontal cortex	Chrna7 KO → ↑ depression-like behaviors, ↑ inflammation, ↓ synaptic proteins in mPFC. Subdiaphragmatic vagotomy → reversed depressive behavior and altered microbiota composition (↑ *Lactobacillus* spp.).	Chrna7 deletion → gut dysbiosis + systemic inflammation → affects brain via vagus nerve. Subdiaphragmatic vagotomy → modifies gut–brain communication → normalizes behavior and brain protein expression	[144]
7	Combined human gut microbiota data from 410 individuals with mild cognitive impairment or Alzheimer’s disease, and a rat model of memory impairment induced by pharmacological parasympathetic suppression	Scopolamine was injected in rats at a dose of 2 mg per kg of body weight to suppress parasympathetic nervous system activity over six weeks, combined with a high-fat diet.	Suppression of the parasympathetic nervous system → associated with altered gut microbiota in both humans and rats → increased abundance of *Blautia*, *Escherichia*, *Clostridium*, and *Pseudomonas* in memory-impaired groups → reduced *Bacteroides* and *Bilophila*	Parasympathetic inhibition via the vagus nerve → contributes to gut dysbiosis → affects cognition by disrupting gut–brain axis communication, potentially facilitating the progression from mild cognitive impairment to Alzheimer’s disease	[83]
8	Zebrafish fed omnivorous, herbivorous, or carnivorous diets; gnotobiotic larval zebrafish model used for microbiota intervention	Dietary intervention with specific feeding habits; administration of *Cetobacterium somerae* and acetate supplementation; glucose and insulin assessments	Omnivorous and herbivorous diets → ↑ glucose homeostasis, ↑ *C. somerae* abundance. *C. somerae* administration → ↑ insulin expression and improved glucose regulation. Acetate supplementation → mimicked these effects	*C. somerae* → ↑ acetate production → activates parasympathetic signaling → improves glucose homeostasis via a microbiota–brain–pancreas axis	[102]
9	Germ-free mice and conventional mice exposed to insulin-induced hypoglycemia to assess stress hormone responses	Induction of hypoglycemia using insulin; measurement of plasma and urine catecholamines; cecal short-chain fatty acid analysis; adrenal gene expression profiling	Absence of gut microbiota → ↓ baseline and stress-induced epinephrine levels, despite normal corticosterone and glucagon responses. Germ-free mice → delayed expression of adrenal stress-related genes	Lack of microbiota and short-chain fatty acids → impairs sympathoadrenal signaling → reduces epinephrine synthesis and release during stress	[62]
10	Young adult C57Bl6 male mice treated with antibiotics to deplete gut microbiota, with or without recolonization; tested for stress response to insulin-induced hypoglycemia	Broad-spectrum, non-absorbable antibiotics in drinking water for two weeks; insulin injection to induce hypoglycemia; SCFA supplementation; fecal microbiome profiling via shotgun sequencing	Antibiotic treatment → ↓ gut microbial diversity, ↓ short-chain fatty acids → ↓ baseline and stress-induced epinephrine. Recolonization restored microbiota but not epinephrine response. SCFA supplementation → partially restored stress-induced epinephrine release	Gut microbiome depletion → ↓ SCFA signaling → impairs sympathoadrenal epinephrine release, while parasympathetic and HPA axis responses remain intact	[63]

The arrows indicate the direction of change: ↑—increase/enhancement; ↓—decrease/reduction.

**Table 2:** Evidence for the modulation of the microbiome and its functional role in the pathogenesis and therapy of chronic diseases.

	Disease/Models	Study Design and Conditions; Intervention/Dosage	Key Findings	Mechanisms/Microbiome–ANS interaction	References
1	Major depressive disorder (MDD)/human (in vivo, clinical trial)	Randomized, placebo-controlled human clinical trial with 45 MDD patients over 4 weeks. Evaluations included HDRS-24, MADRS, BPRS, GSRS, and serum biomarkers (cortisol, TNF-α, IL-β)	Daily oral administration of *Bifidobacterium breve* CCFM1025 (10^10^ CFU) vs. maltodextrin placebo	CCFM1025 significantly improved depressive and gastrointestinal symptoms; it reduced serum serotonin turnover and modulated tryptophan metabolism; these changes were associated with increased alpha diversity and shifts in microbial composition, implicating gut microbiota–serotonin pathway interactions via the gut–brain axis	[207]
2	Major depressive disorder (MDD)/human (in vivo) and mouse (in vivo)	Combined human clinical trial + chronic stress-induced depressive mouse model. 16S rRNA microbiome analysis; clinical evaluation via HDRS, MADRS, BPRS, GSRS	3-strain probiotic mix: *B. breve* CCFM1025, *B. longum* CCFM687, *P. acidilactici* CCFM6432 (freeze-dried, daily for 4 weeks)	Multi-strain probiotic reduced depression and GI symptoms more effectively than placebo in humans; confirmed psychotropic effects in mice. Serotonergic system modulation identified as the primary mechanism. Likely involvement of vagus nerve and microbial-derived metabolites	[208]
3	Functional gastrointestinal disorders with or without generalized anxiety disorder/human (in vivo)	Observational study (125 participants). Gut microbiota analyzed by 16S ribosomal RNA gene sequencing. Psychological traits measured using validated questionnaires (e.g., Hamilton Anxiety and Depression Scales, Toronto Alexithymia Scale)	No intervention	Patients with both conditions had higher levels of *Clostridium*. *Haemophilus influenzae* was elevated in those with gastrointestinal symptoms only. Microbiota patterns were linked to emotional and personality traits; increased *Fusobacterium* and *Megamonas* were associated with difficulty identifying or describing feelings, neurotic personality, and negative views of illness, suggesting a gut–brain interaction	[209]
4	Autism spectrum disorder/human (in vivo)	Non-randomized controlled study in children (30 with autism, 30 neurotypical). Gut microbiota assessed via metagenomic sequencing; serum metabolites analyzed by liquid chromatography–mass spectrometry	No intervention	Children with autism had lower microbial richness, altered microbiota (e.g., decreased *Faecalibacterium prausnitzii*, increased *Veillonellaceae*), and disrupted amino acid metabolism (low ornithine, high valine). Microbial shifts affected metabolic pathways such as galactose metabolism and the peptides/nickel transport system, potentially influencing brain function through altered gut–brain signalling	[4]
5	Autism spectrum disorder/human (in vivo, open-label clinical trial)	Open-label study of microbiota transfer therapy in children with autism. Plasma and fecal metabolite profiles were analyzed before and after treatment using mass spectrometry	Intensive fecal microbiota transplant (microbiota transfer therapy)	Children with autism showed altered plasma metabolites at baseline (low nicotinamide riboside, high caprylate); microbiota transfer therapy shifted plasma metabolite profiles closer to typically developing children; changes in microbiota influenced systemic metabolism, especially nicotinate and purine pathways. Lowering of p-cresol sulfate correlated with reduction in *Desulfovibrio*, supporting gut–brain metabolic interactions	[210]
6	Autism spectrum disorder/human (in vivo)	Shotgun metagenomic analysis before and after microbiota transfer therapy; 10-week and 2-year follow-up	Microbiota transfer therapy (fecal transplant)	Increased beneficial microbes (e.g., *Prevotella*, *Bifidobacterium*); improved microbial gene function; restoration of folate, sulfur, and oxidative stress pathways; lasting gut–brain effects	[211]
7	Alzheimer’s disease (early stage)/human (in vivo)	Randomized controlled trial, 51 participants with mild cognitive impairment or early dementia, 20-week duration	Intensive lifestyle changes: plant-based diet, exercise, stress reduction, social support	Improved memory and daily functioning; reduced cognitive decline; increased plasma beta-amyloid 42 to 40 ratio; lifestyle changes improved brain function and gut microbiota, suggesting modulation of the gut–brain axis and amyloid processing	[212]
8	Alzheimer’s disease risk (APOE genotype)/human and mouse (in vivo)	Comparative study using fecal microbiota sequencing and metabolomics in humans with different apolipoprotein E genotypes and in transgenic mice with human APOE genes	No intervention	Specific bacterial families (e.g., *Prevotellaceae*, *Ruminococcaceae*) and butyrate-producing genera varied by apolipoprotein E genotype; differences confirmed in mice; apolipoprotein E genotype influences gut microbial composition and metabolic output (e.g., short-chain fatty acids), suggesting a link between host genetics, gut microbiota, and neurodegenerative risk	[213]
9	Pre-diabetes/human (in vivo)	Randomized 6-month trial (*n* = 200). Comparison of Mediterranean diet vs. personalized postprandial-targeting diet, with gut microbiome and metabolic monitoring	Personalized diet based on predicted glucose response vs. standard Mediterranean diet	Personalized diet led to greater microbiome diversity and better clinical outcomes (e.g., lower hemoglobin A1c, improved blood lipids); specific gut microbes mediated the link between diet and metabolic improvements, supporting microbiome-driven personalization of nutrition	[214]
10	Rheumatoid arthritis/human (in vivo)	Randomized trial with 22 patients. Comparison of two treatments over time; gut microbiota assessed before and during therapy	Herbal formula Huayu–Qiangshen–Tongbi with methotrexate vs. leflunomide with methotrexate	Both treatments improved symptoms, but gut microbiota changes were more pronounced with herbal therapy (e.g., *Clostridium celatum* increased); treatment altered gut microbial species and pathways linked to inflammation, including vitamin K2 biosynthesis and immune-modulating metabolites	[215]
11	Inflammatory bowel disease (Crohn’s disease or ulcerative colitis, quiescent stage)/human (in vivo)	Single-blind randomized trial in 52 patients with ongoing gut symptoms despite disease remission; duration: 4 weeks	Low FODMAP diet vs. control diet with dietary guidance	More patients on the low FODMAP diet reported symptom relief and improved quality of life. No significant change in inflammation markers; the diet reduced beneficial gut bacteria (*Bifidobacterium adolescentis*, *Bifidobacterium longum*, *Faecalibacterium prausnitzii*) without affecting microbiome diversity or systemic inflammation	[216]
12	Multiple sclerosis/mouse and human (in vivo)	Experimental autoimmune encephalomyelitis model in mice; pilot trial in humans with multiple sclerosis	Intermittent fasting/intermittent energy restriction	Reduced disease severity in mice; improved immune and gut microbial profiles in both mice and humans; increased microbial diversity, enhanced antioxidant pathways, reduced pro-inflammatory T cells, and protective effects transferred via fecal microbiota transplantation	[217]
13	Metabolic syndrome/human (in vivo)	Randomized clinical trial, 39 adults aged 30–50. Duration: 8 weeks	Modified intermittent fasting: 2 days/week with ~69% calorie reduction	Reduced fat mass, oxidative stress, inflammation, and improved vascular function; intermittent fasting altered gut microbiota composition, increased short-chain fatty acids, reduced lipopolysaccharides, and shifted carbohydrate metabolism pathways	[218]

## Data: Availability Statement

No new data were created or analyzed in this study. Data sharing is not applicable to this article.
